# Supplementary material for: “It’s behaviors, not identity”: Attitudes and beliefs related to HIV risk and pre-exposure prophylaxis among transgender women in the Southeastern United States
Source: PLoS One. 2022 Jan 27;17(1):e0262205. doi: 10.1371/journal.pone.0262205 (PMC8794203; doi:10.1371/journal.pone.0262205)
Supplement: S1 File — (DOCX) [file pone.0262205.s001.docx]

Van Gerwen

HIV Study

File Name: File 1-Audio Only

Length of File: 65:52

Mod: I have a list of a couple of questions that I want to use to guide our conversation but really the goal is here is to hear from you, to hear your perspective on these things. So even though I came up with a list of questions, if these aren’t the right questions, if these aren’t the things that you think I need to be asking about, tell me that and let’s lead it in the direction that you think we need to go. Because the goal here is really to figure out, you know, some of these issues from your perspective, right? When we… you know, when we think about how we can better serve different communities one of the critical things that we do is try to hear from those communities… you know, what’s working for you… what isn’t working for you? And so that’s why we’re all here today and that should give some context to the types of questions that I’m going to be asking today. You’ll notice that a lot of the questions that I ask today talk about community, and I’m going to say often times, you know, tell me from the perspective of your community. And part of the reason that I say that is that I don’t want people to feel like you’re revealing your personal, deepest, darkest secrets, right? Even though we have confidentiality here, even though we’ve all promised that everything we say here is just going to stay here, I do want you to feel free to say, well, you know, I might think about it this way but I’ve heard some other people say this, as well. Part of the reason for that is that helps us to have even more perspective here. So right now we only have one, two, four, five… five people here, but if you’re able to say, you know, I hear a lot of people say or I’ve seen on message boards people say, you know, such and so, that’s really helpful, so feel free to bring in information that you’ve heard from other people in your community and their perspective on these issues, as well. So, does that make sense?

[Several affirmative responses]

And one of the things I’m going to do… and if you’re… if you’re hugely uncomfortable with it and you just want to say… that’s fine. But just to make sure that we’re hearing everyone’s voices I may occasionally call on you and please don’t feel like you have to say something… ohh!... you know, like you’re put on the spot and if you don’t (02:10-inaudible-recording breaking up) still thinking about it or just, you know, (inaudible). But I do want to make sure that everyone gets an opportunity to share their thoughts on these things. So I will do a little bit of moderating, but not too much. My hope is that we can just kind of have a free flowing conversation about some of these things, talk through the things that you think is important. So does that sound good?

[Affirmative responses]

Okay. And everyone can hear me? You can? Okay, good. So the first thing we want to talk about, and as you know from what Victoria told you about the study we’re really interested in HIV and how people think about HIV. So the first thing that I’m interested in is just that. Tell me about HIV in your community. Is HIV a concern? Is it something that people talk a lot about or think a lot about? Go.

R: For me, it’s not much of a discussion unfortunately. Um, I’m not sure as to why but it’s not something my friends and I we often talk about. I mean we’ve had discussions about it before but it’s not… we will lightly talk about it and then we’ll move on to something else. So it’s not, yeah, very discussed from my space.

Mod: Okay. What do others think?

R: So I’ve never really actually like had to deal with HIV or any of that stuff because I’ve only dated, you know, people that have always… you know, not high body counts and stuff… I’ve always been sure to like ask them what their body counts are and stuff, so.

Mod: Okay. What do others think? Do people see risks for HIV?

R: I haven’t really thought about it that much either. I was always more worried about STDs. I didn’t even know apparently there’s a drug that stops HIV now that they give to some of the trans women sometimes.

Mod: Oh, yeah, we’re gonna talk about that.

R: I had no idea like that drug existed.

Mod: Yeah, yeah. Okay. That’s interesting that you say you do think about STDs but you don’t really think about HIV. I wonder why that is. Do you have thoughts on why you think about one but not the other?

(04:41-No responses)

Is that an experience other people have had, of saying, yeah, I mean I think about, you know, gonorrhea, or maybe syphilis, herpes, but I don’t really think about HIV?

R: I know in my specific case a lot of it has to do with being in a much more conservative circle, so there’s very, very little talk about HIV but there usually is a lot more spent actually discussing STDs, which I’ve never thought was weird until recently.

Mod: Interesting. This is just fascinating to me because I’ve done studies where we’ve tried to really focus on STDs and everyone always only talks about HIV. So I’m hearing kind of the flip here and I’m fascinated by that and what could account for that. So any other thoughts about feeling about being at risk for HIV?

R: Um… well, I have a very similar experience to the first person that talked, but I do, uh… the main places that I’ve talked about HIV and have been… I go to Auburn University and I attend (06:06-inaudible) from there just through Christian Alliance and so we held talks about like LGBT health a lot and that’s where I’ve mainly gotten all of my information. But if I didn’t go there… and you’d be surprised how few people actually go to the GSAs, especially in a campus with a climate like Auburn’s.

Mod: Yeah.

R: There’s like 20,000 students but there’s only like twenty people there. So, yeah.

Mod: Interesting. Okay.

R: I also…

Mod: Oh, yes… I’m sorry, who’s…

R: It’s the same way. That’s how I feel, too, because I was like in a small town and like not a lot of people really accept me or, you know, all that stuff.

Mod: Uh-hum.

R: It’s hard like growing up in a small town.

Mod: Yeah.

R: A lot of information that I get tends to be internet-based and I feel like a lot of focus kind of pivoted away from HIV. Granted there’s a lot just going on, so there hasn’t been a whole lot of just general discussion. So without… without Twitter talking about I don’t really have a lot of ways to get access to the information.

R: Hi, how y’all doing? Hey, I’m [name redacted]. My pronouns are she/her/her. Um, I’m glad I could be a part of this Zoom with you guys. Um, I’m actually a HIV prevention specialist and a former PrEP navigator here in Birmingham, Alabama, so I know about linkage to care and (inaudible) case management and things adults… I hear people that are on here now, I hear you guys claim that you’re having a hard time finding information. Well, if you ever come to Birmingham, if you’ve ever heard of Birmingham Aids Outreach, um, even though we’re doing the Covid situation they do offer confidential HIV and STI testing. They do HIV 101 education, as well. They walk you through the whole process and let you know that you’re safe, everything is confidentiality-focused and also we have our mega-city acceptance center that’s open to LGBTQ youth and adults, as well, and they offer supportive services and linkage and different things that’ll help you focus on learnin’ more about HIV and testing and stayin’ safe and stuff like that.

Mod: Awesome. What a great resource to have them call. Um, yeah, I love that you’re on the call, [name redacted], and you’re gonna give some great… some great ideas. So thinking about other spaces… you mentioned Twitter… thinking about other spaces, you know, before we started some of you were talking about when you started transitioning or even things like that. In trans communities do you hear a lot of talk about HIV or is the focus on something different? What do you think?

R: The focus in all the trans communities that I’ve ever been a part of has strayed from physical health and much into mental health. If we’re ever talking about our health it’s generally like mentally coping essentially.

Mod: Interesting! Oh, that’s a great… that’s a great insight, yeah.

R: And from the standpoint of a trans woman of experience as far as an African American community you don’t hear the talk of HIV. The only time you do hear the talk of HIV is when girls are Spanish (what it sounded like to me) shaming. And there’s Spanish shaming… when I say Spanish shaming is talkin’ to give someone else’s status and not knowing their own. There’s a lot of talk about self-care. There’s a lot of talk about mental health and there’s a lot, a lot, a lot of talk about (10:10-inaudible).

Mod: Uh-hum.

R: So I feel that it should be a conversation that we put on the floor and opened up for you and your good girlfriend or you and your boys, y’all together, just open the conversation of, you know, how well are you takin’ care of yourself. And when a person can’t sit up and say, oh, I’m clean, clean could be I just washed my hands or I just washed by face or brushed my teeth. That’s not defining if your status is negative. But a lot of times we overlook that because we’re more focused on… and a lot of trans women I find are more focused on the look, it’s always about the look. And you have to sit back and think what does trans really look like. Is there a look for tran? Is there a look for a cisgender white man? If we take the focus off of how we look and focus on our inner selves our outer look will come together… it’ll come together way easier because you’ll know that one, my health is here, I’m focused on it. You know, a lot of girls stray away from getting HRT services because they’re scared of takin’ that test, they’re scared of that bloodwork, you know. But we can’t be scared. We can’t live in a world where we have risky sex and unprotected sex and be scared to go to the doctor. It doesn’t add up.

Mod: Yeah. So I’m hearing you say that… that maybe part of the reason that HIV isn’t a big thing discussed is because there’s other things, particularly mental health, that are more of a focus, that people talk about more. Does that sound…

R: We don’t have HIV advocating in the trans community. When they present HIV, and this is not to discredit any type of grant or CDC funded thing, it’s always geared towards men who sleep with men. We have to take the focus off men who sleep with men. There’s women who sleep with men, too. There’s trans women who sleep with men. It’s not always (12:20-inaudible)

Mod: (inaudible) muted… Unmute yourself.

R: You’re muted.

Mod: Yep, unmute.

R: Uh-oh.

Mod: I don’t think I have… I’ll be able to unmute.

R: … unmute her. I think she may have lost her connection.

Mod: Oh, okay. Yeah, because I tried to unmute her from… okay, well, we’ll… hopefully [name redacted] will be able to…

R: I’m back.

(laughing)

R: I’m sorry, you guys. That was my groomer. My dog is on the way up here so I had to get my dog.

(laughing)

Mod: Okay, so you were saying… and I think this is a really important point, that part of the issue is that a lot of prevention and messaging is really focused to specific groups and it’s not focused to trans women, but it’s focused primarily on men who have sex with men.

R: Yeah.

Mod: And so that… okay, so do you think that, I mean in general when people think of HIV who do they think of?

R: Men who sleep with men.

R: Yeah, like every ad I’ve ever seen it’s like…

R: Every gay man gotta have HIV. Every gay man gotta have HIV. Every trans woman gotta be a prostitute or a sex worker or some type of sex dealer. There’s too many stereotypes and we have to break those stereotypes, all those different biases that people sit up and put us under these umbrellas. Those umbrellas are not getting respected or acknowledged.

Mod: Okay.

R: You understand?

Mod: Yeah.

R: It’s always… oh, well, he’s gay, he’s gay, he’s gay, he’s gay, he have to… no, it’s the stereotype and the stereotype plays back to where when you… that’s when people go and… they go off and they live in (14:15-inaudible) and they don’t wanna be a part of the community because the community’s always pointing fingers. But I’m a believer that when you point one finger you got three pointing back at you.

Mod: That’s true, yeah. Okay. So it sounds like…

R: Y’all talk and I’ll be right back.

Mod: Part of the issue is that maybe the messaging isn’t there equally for all groups. Does that kind of… how does that strike other people?

R: I agree.

R: Right. Like any time I see any HIV ads on Facebook it’s always men and men, nothing geared towards me or transgender folks at all.

Mod: Okay.

R: I’ve kind of noticed that in my groups with other trans people and stuff, we tend to talk more about legality stuff, and the mental stuff, too, but like, you know, name change issues and people… like work discrimination… that comes up a lot more for me because most of the other trans women in groups or people that I know we’re not super sexually active to be honest with you. So like… like I don’t… haven’t even thought about since I started this, like I’m sure it’ll come back, but it’s just not on my radar like at all.

Mod: Interesting. Okay, so I’m hearing a couple of things. I’m hearing, and several of you have said that it sounds like of all the issues that you could be talking about with other trans women there’s a lot of other issues that are just more important, mental health, legal issues, transitioning, these sorts of things. And then I mean HIV would be maybe way down at the bottom.

R: For me. I know with other communities, but just the people that I know.

Mod: Okay. But then I also heard you say that right now it’s not really much of issue because, you know, in any sort of relationship that would put you at risk for HIV. And I think that’s really key, you know. We know how people get HIV, we know how HIV transmission works, and so if you know that you’re not engaging in behaviors that could potentially expose you to HIV maybe it’s just not that big a concern. I’m curious what others think about that because I honestly hadn’t thought about that. Does that square with other people’s experiences?

R: Well, for me, I’ve remained sexually active just by being on hormones. I mean the physical effect is obvious because estrogen lowers your libido, but I… the reason that I haven’t been particularly worried about HIV is because it… as a trans woman I haven’t gotten a lot of safe sex talk but as a left wing person I happen be lucky enough to receive some and I generally associate in a community that has been lucky enough to do that as well, so that’s my reliance, I rely on that.

Mod: Okay. So when you say you associate in a community do you mean like the broader queer or LGBTQ community or different communities that you connect with?

R: I will admit I come from a sort of an affluent background at least. I’m from Batavia Hills… it’s, uh…

R: Okay, I’m from Leeds.

R: Yeah, some people aren’t like… when I moved to Auburn I knew a lot of people that come from towns with graduating classes with like a hundred people and they’ve rarely seen a condom.

Mod: Okay, so you think a lot of it’s kind of contextual in how you grew up, you’d already heard a lot about HIV?

R: Yes.

Mod: Okay. So any other thoughts, anything else? This has been really… this covered a couple of really important things here already in thinking about how people… how people think about HIV risk generally, who we tend to think of as being at risk for HIV, and sort of how HIV stacks up in terms of other things that people want to talk about, other concerns that people have that they tend to talk more with friends about. Are there any… any other big things about HIV and how people think about their own risk for HIV, especially trans women? Are there any other big things?

R: I definitely wish there was more education about it. Like growing up I didn’t receive like any education on HIV whatsoever. And just in my adult years I’ve had to do my own researching, reaching out to other people, learning from them and just learning from Google about HIV. So I wish there was a lot more education on it through the public.

Mod: Okay. When you search for information… when you’re trying to get, you know, good, reliable information how do you find that on the Internet?

R: Usually I’ll go to YouTube to folks who are transgender who I like, who I trust, I know their story, their background, them. Then I’ll just talk to some of my friends who are also transgender and then just Google.

Mod: Okay. Others? What are other sources that people have gone to for information specific?

R: Um, my doctor, when I… when I finally did get the chance to… to have… to get on HRT I finally was talking to my doctor about AIDS, but it wasn’t until then I actually had it been brought up by my doctor at all. So that was an informed consent clinic, so it was actually was important (20:01-inaudible).

Mod: Okay.

R: Uh, so when I started HRT my doctor asked me if I was sexually active and I told him that I was not sexually active because I wasn’t, and I’m still not sexually active because I’m actually in the process of moving my scene, so, um, HIV’s never been like a huge like worry in my life, I guess because I’ve never really been sexually active with people. Um…

Mod: Okay.

R: So…

Mod: No, that’s… that’s good insight, that’s… anyone else want to share before we move on to our next topic?

R: Yeah. When I started my HRT it was a joke… it was a joke. I am a plus, plus size girl and they thought they were gonna start me off with two milligrams. Anything that I asked for it was a no. I had to advocate for myself. I had to challenge them and I had to let them know that my transition was very, very serious and it was very detrimental that I get the things that I needed. I then transferred my HRT to… you guys heard of (inaudible), this place out here in Birmingham, Alabama. They’re very (21:35-inaudible). My endocrinologist gives me no problem; he works with me, anything that I ask him for or information, being that coming from a background of black market silicone and black market hormones I knew my limits. You know your own body. Yes, the doctor is there to help you when it comes to your own body, but you must advocate for your own body ‘cause you know how you wanna look, how you wanna feel. And them two milligrams were making me stressed out and I wasn’t seein’ any progress so ended up upping my doses and anytime that you feel that they’re spoon feeding you… that’s what we call it… spoon feeding… you have to stop ‘em in their tracks ‘cause they’re gonna get rich and you’re still gonna be sittin’ there in misery. Your transition’s basically gonna be null and void.

R: Exactly.

Mod: Um, okay. So anything else with regard to the things that we’ve been talking about… about HIV, before we transition to our next topic? Anyone else wanna jump in here?

R: Um, last thing would be like if I hadn’t lived around the Birmingham area, so like a really big city with resources in it, I don’t know if I would like have the opportunity… because I go to the MCAC, too, or I go to the Magic City Wellness Center for…

Mod: Okay.

R: Yeah.

Mod: Okay. So just having access to providers?

R: Yeah, and it’s very dependent on being close to a large city.

Mod: Alright. Okay. So this has been really helpful, thinking through HIV. I want to shift a little bit now and talk about something that we touched on a little bit and that is the idea of PrEP. How many people have heard of PrEP?

R: I have.

Mod: Cin, I’m guessing you heard of PrEP?

R: Yes.

Mod: Alright, so when we’re talking about PrEP we’re talking about Pre-Exposure… prophylaxis, right? And right now that can be done in one pill that’s taken once a day, right. So this is an option. And I’m curious what do you guys hear? Where do you hear about things about PrEP and what do you hear and what are some of the perceptions about PrEP among trans women?

R: I really don’t know much about it; I just know that if you’re going to be sexually active you should probably get on it, and that’s literally all I know. Um, I don’t know anyone personally who’s on it. Um, but I mean I’ll see dating apps where… or more specifically, just Grindr, where they’ll say they’re on PrEP, but other than that it’s not really discussed, Myspace.

Mod: What do you think when you see that on Grindr? Do you (inaudible) or do you say like, oh, yeah, that’s great, or oh, that’s weird?

R: For me, I’ve heard a lot of good things about it, so I just imagine they’re just being really responsible with their body and they’re doing what they’re doing.

Mod: Uh-hum. What do others think? Have others seen that on like apps, dating apps or…

R: Um, I’ve not seen PrEP on dating apps, but the first time I ever heard about PrEP really was when I went Magic City Wellness Center. Nothing before that. I just knew it was like related to AIDS before that point.

R: I saw the commercials, didn’t really know what they were talking about and I… one of my friends who’s trans, she’s actually African-American, too, came over and we were talking and she’s apparently on something like that and that was the first time I heard of a trans person being on it. She talked about expensive it, whatever she was on was, and if she didn’t have insurance there’s no way she could afford it.

Mod: Hmm… uh-hum. When you say you saw ads for it, like where?

R: There’s like ads on… they used to come up… I guess they stopped paying for it, but it was full of gay guys and they would like talk about PrEP and stuff and I didn’t really know what it was.

Mod: So that goes back around to that idea that it was targeted at the specific… at specific groups. Okay.

R: (inaudible) dating app, it’ll market PrEP, too.

Mod: Kelly, is that you?

R: Yeah.

Mod: Uh, speak up. I can’t hear you.

R: If you sign up for like an LGBT dating app, then like that’ll be saved on like your personal profile and then advertisers will market PrEP to you.

R: But the promotion for PrEP is not promoted to trans women or trans men; it’s promoted for men who sleep with men. So once again we have this barrier that… that are being put up and being disconnected and dis-included and it’s making girls think, just not thinkin’ about. That’s one reason girls are not trying to get on PrEP and also the stigma of this is an HIV medication. I’m HIV-negative, so therefore why would I wanna take something? In the back of a person’s mind it’s like, well, I’ll take it so long and what if I contract HIV… what if the medicine does not work because everybody’s immune system is totally different. So just because a doctor gives me this particular type and I take this does not block me from contracting the HIV virus. It also (27:31-inaudible) never get the full detail about PrEP. They talk about that it’s the drug for HIV, but they need to specifically let people know that this is not a magic pill. This is still something that you still use protection and also it does not protect you from any STIs or STDs. And if you contracted an STI or STD you (27:56-inaudible) for HIV or any other virus or disease that may come your way. PrEP is not discussed in the trans community; it’s more so discussed in the man to man community. Sorry about that, y’all… you can hear outside. I’m still at my job at work. And it’s not discussed; they don’t ever set up events geared towards the trans community to get involved when it comes to PrEP. They just now created this commercial to include trans people of what… like I said earlier, of what they think trans look like. But not discrediting anybody in the (inaudible) commercial, there is only one trans woman in that commercial, it’s not two, and people get it mixed up. They get the difference between cross dresser, drag queen and trans woman or transgender totally confused.

Mod: Okay.

R: And we’re never invited to the table when it comes down to talk about PrEP unless you have a PrEP navigator like myself, a navigator that will (29:20-inaudible) women knowin’ that my target is basically for men but I still want to be open and available for anyone that might wanna get it can.

Mod: Okay. So I’m hearing lots of things again with sort of targeting and even when things are targeted like the commercial that you noted sometimes it’s not targeted quite right. My guess would be that maybe they didn’t talk with people in the community about what would be the best way to represent different people’s experiences. Also, I heard you talk about stigma, and that’s something that I’d love to hear some other thoughts on, too. [name redacted], you specifically mentioned the stigma associated with… well, why would I be taking an HIV medication if I don’t have HIV. And what if people see me taking HIV medication and they get the wrong idea. I want to hear other people’s perspectives on that and on other barriers that you can see, particularly for trans women who are thinking about taking PrEP. We’ve already said it’s not well-known, but once people hear about it why do you think more people don’t take it?

R: Maybe because they’re ashamed of like what other people will think about them. People may judge them for taking it because like stereotypically like… like PrEP is stereotypically meant for men who sleep with other men. So like trans women may feel as if they’re not meant to take it because they’re women, you know.

R: I guess I would be confused… well, I am actually as to why some people should be on PrEP and not other people when everybody in the world has sex. I don’t really understand that difference.

Mod: Okay, so maybe it sounds like the messaging around who should be on PrEP, how you should think about whether you as an individual need to be on PrEP, maybe that messaging isn’t coming through very clearly, it sounds like.

R: Yeah, I still don’t… I still don’t understand like is there some particular reason I should worry about it that another human being that also is sexually active is less worried about? I mean I don’t get that.

Mod: Okay. What do others think? This… that’s… I think that’s some good insight.

R: Um, personally the reason that I don’t use PrEP is it’s a mixture of little things that we talked about lately. I do tend to be with people that have lower body counts but also I rely on them, on the safe sex practices. But the reason I didn’t end up doing PrEP is it was… I think it was a good time to introduce it to me when it was but the reason I didn’t take it was because of the side effects, or I was worried about the side effects, while I was dealing with a transition. Yeah, that was the main reason.

Mod: Okay, so concern about side effects. Were you particularly concerned about because you might already be having some side effects from transitioning and then layering that on?

R: Yeah.

Mod: Afraid there were gonna be like interaction like between…

R: Yeah. Not like medically… a lot like mentally, like….

Mod: Oh, okay. No, that’s an important… that’s an important distinction.

R: Yeah.

Mod: I mean medically, you know, there may or may not be, you know, just depending, but that’s… that’s a bigger concern I think, sort of the mental aspect of, you know, this is another thing that I have to be thinking about. Okay. Very… very helpful.

R: And from my kind of perspective it’s more of a stigma issue, that HIV is still very heavily stigmatized versus other STDs like… and super conservative background but I at least heard about STDs and generally safe sex practices. Not very well, but at least some. So I feel like there’s probably still a really big kind of like if you have HIV it’s your own kind of fault like mindset in regards to that. I don’t really know how we’re supposed to go about changing that but I feel like that’s kind of a more prevalent thing.

Mod: Yeah… no, I mean that continues to a big… it’s a big stigma.

R: I can also only…

Mod: Yes, uh, what were you going to say?

R: Also, I don’t have insurance. I’ve never had insurance except for my parents’ insurance, so anytime you add medication that doesn’t have to do with my transition technically I’m gonna be less willing to pay for it because I don’t have a lot of money.

Mod: Yeah. No, I mean that’s absolutely a consideration, uh, that (34:45-inaudible)… yeah, [name redacted], what do you think?

R: I always suffer from high blood pressure and my mom had a renal failure to hear those side effects as far as kidney failure and also the fact that I’m on heavy HRT medication, also, you can’t really take alcohol with medication. And I know that I’m an entertainer and I know the couple times that I go places that I drink and I have more than one or two drinks, so just thinking about me constantly taking that medicine it scared me because I know there’s day where I might wake up and I might be like late for work or on a high and in the hustle and bustle of things I couldn’t even take those meds even if it is one dose a day, and they don’t want you to miss it. And I don’t wanna mess up my body on the inside taking this drug, being inconsistent and getting off track and then trying to get back on and then the list of things that could happen… it’s a lot of underlying things that play a role in it. I think it should be promoted in another light, another way. Maybe if it was promoted from a, I would say like a cis point of view, like if there were sex workers that wanted to get on it and it was cost efficient and they didn’t have to go to (36:00-inaudible) and they didn’t have to have insurance and Gilead gave them a co-pay card. Something should be… I understand it’s an expensive drug but even though I know they do trial studies the majority of the time those trial studies are not geared towards straight people.

Mod: Okay. So you’re saying maybe… maybe we need to think about targeting groups that are really… ‘cause, you know, you’re saying for you, you know how to assess your risk and for you you’re more concerned about the potential side effects of taking the medication. And for you, you’ve done that calculation, and you think for you it makes sense not to take it. But you’re saying other groups, their calculation of their risk versus how they weigh the side effects is gonna be different. That’s… that’s really interesting. So thinking about who we… how do we tell people that? [name redacted], I know you’re, you know, a… you… you know, give health education, but I’m curious to hear from others who don’t, you know, who don’t do that for a living. What is… how do you want to hear about these things? Everyone said, oh, well, kind of heard about it, and, well, I may have heard it from my doctor or a couple of people talked about seeing commercials for it.

R: A doctor.

Mod: What would be the ideal way to get information about PrEP that would help you to make an informed decision about whether or not it was right for you?

R: My doctor.

R: Well, I feel like if you’re gonna go get checked at like an STD clinic that they should inform you that, hey, if you’re really sexually active like maybe you should try PrEP, you know. Like just spread the word.

Mod: Okay, so specifically around other sexual health stuff.

R: Uh-hum.

Mod: So a doctor or like at a… at a testing site. Okay, that makes sense. Other thoughts? What about… Like should we make a big billboard?

R: Yeah, like

(38:32-Several talking over each other-inaudible)

Mod: Who’s into the billboard? Is that… is that…

R: Focus group.

R: Really I think that really is a good idea because when I’m driving home I read billboards, too, so I mean that would be a really good idea.

Mod: Okay, I was kind of joking about the billboard but okay. We’ll get you over to make a billboard after this. (laughs)

(laughing)

Mod: Other… other things… I won’t mention my side of the bus idea because I know you’re gonna be all over that one, too. Have you ever seen the big ads that they put on the side of the bus?

R: Yeah.

Mod: The advertisements? Do we need one of those?

R: Do we have buses?

R: And also find someone that has a… a good following of people.

Mod: Ahhh.

R: Yeah, like an LGBT…

R: You know, like an LGBT influencer.

R: Like (name) or like Nicky the Dragon, or somebody like that.

R: Right.

(several talking over reach other)

Mod: Okay, this is fascinating, so (39:37-inaudible). Does this influencer, does it need to be someone that you actually know, someone who’s in your community?

R: For me, yeah.

R: Uh-hum.

R: I’m like very much into watching the same person or persons.

R: Yeah, because I feel like if I’m following someone I like it’s gonna make me wanna use it even more.

R: Right.

R: Yeah.

R: Yeah.

Mod: Okay. Interesting… interesting.

R: Like for me, I love Janet Mock and Laverne Cox and even though they’re not really Instagram influencers I would love to see them talk about it because I trust them.

R: Or GG Gorgeous.

R: Uh-hum.

R: (inaudible) Dragon is my favorite, though.

Mod: This is great. I’ll contact all of these people and get with them right away. (laughs) Um, so, talk about… like if you’re thinking about social media and influencers on social media, would it… does it need to be an influencer or someone you know or could it just be like posts on social media?

R: It could be like…

R: It needs to be someone that’s covered the trans community that when they speak up for the trans community they’re speaking up wholeheartedly; they’re just not goin’ off something they were advocating on.

Mod: Okay. So they have to be that personal… it has to be personal, you have to steer them that they’re speaking their own truth about what they’re sharing. Okay. Oh, that’s really good to know.

R: Makin’ an impact and not (41:24-inaudible)

Mod: Okay.

R: That does help. I find that in addition to Twitter a lot of place where I personally get a lot of information comes from Reddit. So having a… like contacting specific trans sub-Reddits would be a great way to get information out there in a very successful way.

Mod: Yes… yes. Alright, I love it.

R: Or maybe like the PrEP company can like reach out to like influencers and pay them to sponsor.

Mod: Okay, alright, maybe paid stuff… I honestly never think of Reddit but everyone I know reads Reddit all day long, so obviously it must be a pretty… pretty significant.

R: Or maybe like TikTok. Like if they… you know, on TikTok…

(42:20-Several talking over each other-inaudible)

Mod: Okay, and what… I mean TikTok’s powerful.

R: I get a lot of information from YouTube. My favorite YouTuber is ContraPoints. Like she’s a trans woman and…

R: I like her.

R: Yeah, she… yeah, just I… I feel like if we targeted YouTube then like sponsorships would just… that would get a lot of attention.

Mod: Okay. Anything else? This is so… this is so helpful. Other platforms? I mean I’m still on Facebook. Is anyone else on Facebook?

R: No.

Mod: I already knew the answer to that question (laughs). It’s only people like me on Facebook now. It was so cool when it started. (laughs)

R: I use it to socialize but not for information.

R: I have Instagram but I deleted my Facebook because people could find old pictures of me and it just freaked me out so I was like…

Mod: Yeah… yeah. Okay, so maybe a little Insta… Instagram. Okay.

R: Yeah.

Mod: And I know someone said Twitter earlier. Um, but yeah… Reddit. And I don’t… I don’t really think of YouTube… like I go to YouTube for specific things but I hadn’t thought about following…

R: YouTube has got a lot of people that will do like commercials if you pay them and they’ll talk about it before the video starts.

R: Yeah.

R: Like all of… like I don’t have cable, I don’t have… I barely have Netflix. I’ve literally… all of my entertainment is through YouTube, all my information is through YouTube.

R: Right, yeah. I only have YouTube and Netflix and Hulu and that’s all.

Mod: Okay. That’s really powerful. Okay, wow. Oh, you all are giving me really good… really good ideas. So what else? Those are the big questions that I wanted to talk through. I’m curious, based on everything that we talked about, I mean you can see where we’re trying to go. We’re trying to figure out… oh, gosh, you know, what do trans women know about PrEP, what do they want to know, what are barriers, how can we start to address that and then where can we get this messaging out, right? So you see what we’re… what we’re trying to do here. What else do we need to know about that, what are pitfalls? What should we absolutely not do? That’s a good thing to think about.

R: Honestly, I’m kind of with the stigmatization thing, of like… I think [name redacted] said it… it’s pretty sensitive for me… like drag queens and homosexual men community and stuff, not to dis them; it’s just I don’t really like being associated with them. It’s like a thing that I don’t like, so I wouldn’t want to see a combination ad at all ‘cause I don’t… not that they’re not good people or whatever. I just don’t like the public associating me with… I wanna distance from that.

Mod: Okay, yeah… no, that’s… that’s huge insight. I’m glad that you shared that.

R: Personally I don’t mind. I don’t know, I feel like that might be a very variable thing. Like, I don’t know, I’m very involved with the LGBTQ community personally, so like I just… I find that most of my friends(45:45-inaudible).

R: It’s… for me it’s like… it’s not that they’re not my friends, you know, I’m part of these groups… the regular average… if you’re talking to not people that are informed… people that are informed are already informed. That doesn’t matter. If you’re talking to Average Joe and you start mixing all this stuff together they only see the periphery of it, just lump all LGBT together and… and now that I’m a woman I’m LGBT.

Mod: Uh-hum.

R: Though I’m a woman first.

Mod: Yeah.

R: LGBT second.

Mod: No, that’s…

R: Sorry, I don’t want to be lumped in with sexuality because it’s not sexuality, it’s gender.

Mod: Right. That’s… that’s fair, and I mean that’s the debate that’s been going on in the LGBT community for… ever since I can remember. Are we overlaying too many things? So I think that’s a really fair point. What do others think? Are there any other big… big things that we need to know? Big mistakes that we need to be sure to avoid? Anything else?

R: Well, I feel like my mind just blanks out when I wanna say something… I’m so sorry… I’m really sorry (laughs).

Mod: Well, here’s what we can do… if you think of something… you know, if you wake up in the middle of the night, oh, I should have said this when she asked that… I think you all have Victoria’s email address… Victoria, is that true? Victoria still with us? Yes. So…

R: (47:40-inaudible)

Mod: So shoot her an email, so if you think of something or if you’re shy and you don’t wanna say it out loud here… oh, there it is again… (email address given), so take that down and if you think of something, you know, even five months from now you’re like, oh, wait a minute, I just saw this ad and it made me really mad in a way that I wanna be sure to tell that research team so that they don’t mess up in the same way, let us know. That’s… you know… that’s useful for us.

R: I think for me, for ads like as long as its tasteful and you did your homework behind it, like… oh, and it’s presented in a very nice way, um, I’ll click on it. But if it’s something like targeted… if it’s two men I’m not gonna click on it, you know, um, ‘cause I mean I do appreciate a really good, beautifully designed ad and I will click on it I mean I feel like if it applies to me.

Mod: Okay. So the applies to me part sounds like it’s really crucial.

R: Uh-hum.

Mod: But really the key thing is that you can feel…

R: Yeah, if it applies to me.

Mod: Okay. That’s helpful. Other stuff like that?

R: Yeah, either really specifically focused ads per group or if it’s going to be a very just LGBT period kind of ad then very specific focus on quality and quantity of representation in the ad.

Mod: Uh-hum, okay. Alright, so being really mindful, yeah. Yeah. No, I mean whenever I see… whenever I see an ad that’s targeted to the LGBTQ community I’m always… I think we all do, we start counting… oh, okay, well, they’ve got a couple of those people, but they don’t have any of these people and stuff… I mean we look for it.

R: It’s easy for an ad to feel like an LGB ad and not an LGBT+ ad.

Mod: Yes. Well, yeah. Don’t get me started on the B. (laughs) Because B tends to be invisible, too.

R: Yeah, people leave out B a lot.

Mod: Yes, I know.

R: And even like intersex folks and asexual folks, you know, they’re part of our community, in my opinion.

Mod: Yeah… yeah.

R: I know a lot of (50:31-inaudible) don’t like to just be lumped in with… they’re intersex, it’s different.

Mod: That’s… yeah, I mean that’s another huge… that’s another huge debate, is, you know, is there a power in defining things narrowly or is there more power in define things broadly and who do we include, who do… who then gets left out. Trust me, I work with this all day.

R: Different medical issues.

R: Well, I feel like a lot of people feel… ‘cause I’ve heard people talk about this, that like they don’t think like the T should be included in LGBT because they consider T not a sexuality. And I’ve heard a lot of people say that.

Mod: Yeah… yeah… and, you know, I mean there’s historic reasons. So full disclosure, I guess I should have said this at the beginning, I’m actually a sociologist and I used to teach courses on gender and sexuality, it’s my area of research, so when I say don’t get me started I really mean don’t get me started because I could give all sorts of history lessons.

R: That was my favorite class.

Mod: Yeah, it was… it was fun classes to teach except for it got really old hearing, you know, most of the way through the semester and a student would say, oh, I have a cousin who’s gay, and that was like a big revelation for them and I’d just be like what have we been doing all semester if this is your big revelation. (laughs) But that’s just me, so…

R: I know that this is probably not popular with some people but I still really like the term gender minority and sexual minority. I just think it’s easier to say, less confusing for people that aren’t involved in it and make distinctions that I find to be very important.

Mod: I, from an academic standpoint, I concur. Um, yeah, that’s… that’s the terminology that I definitely feel more comfortable when I’m writing about these issues, tend to always use… yeah… the language. Alright, so sounds like we’re winding down. Are there any thing… pressing things that you want me to know? Like I said, you can shoot Victoria an email if you think of something or you have something that you want to share just privately and you can send that to Victoria and you can tell her, hey, send this along to the moderator, [name redacted] or not. So what do you think? Anyone have something big they wanna conclude on? [name redacted], are you gonna say something? You looked like you were taking a breath.

R: Like, um, marketing directly to transgender people is going to be surprisingly difficult because a lot of transgender people, especially in online situations, try not to identify themselves and certain things that I’ve realized. So to have a profile about them to define them as transgender is more than likely not gonna be possible. I mean, but there are private profiles defining them as affiliating with the LGBT and I think if you wanna market something towards transgender people, and this is like a popular marketing tactic, and whether it’s successful or not is another debate, but, uh, (54:15-inaudible) fake profiles to talk about things, so just opening a conversation surrounding it inside of transgender groups and transgender communities and just having that conversation I think is going to…

R: (54:33-inaudible)

R: … because then you get people talking about it and, you know, you kind of break away from the stigma or anything like that because of the openness conversation and then more and more communities is probably a way to reach out to specifically transgender people rather than the community as a whole. Because if you’re just gonna market something it’s just gonna be, you know, are you affiliated with the LGBT but it’s not gonna know specifics of gender, sexuality.

Mod: Got it, and that’s a… yeah, that’s… that’s a really fair point, as well, how people choose to identify. And that changes over time, too, so being mindful of that… and I think that… I mean that pushes us to think even more carefully… who are we really trying to target… who are we really concerned with? Is it identity, how people identify that we need to be concerned or is it just behaviors that people engage in? So a lot of people said, you know, if you’re sexually active you need to be thinking about PrEP; if you’re not sexually active you don’t need to be thinking about PrEP. So really trying to tease that apart and think about all of the issues that y’all have raised tonight about what it means to really try to speak to a particular group of people and, you know, help them to understand whether PrEP is something that’s advisable to them. This has… this has been so… so helpful. Every time I talk to people I just have eyes totally opened, so I appreciate that so much and your willingness to talk openly tonight. Is there anything else that anyone wants to say before we wrap up?

R: Uh, and idea came across me… um, the… the time when I actually started learning a lot more like important information about my own identity…

Mod: Yeah?

R: I mean like was when I started like actually socializing with people like in person, um, and I don’t wanna be like a screen bad but like it… it was something to like be like socializing with a group of people just in person because it was some… I don’t know, there’s something about it, like that changes it.

Mod: Okay.

R: I just learned a lot more that, so like if there were more groups, I mean, sometimes I go to the Magic City Acceptance Center, right, um, and I don’t know if this is even viable but if there were like more groups in Birmingham that like, I don’t know, find people who were more like you then that would be…

Mod: Okay, so I’m hearing you give a plug for peer-to-peer kind of organizing… something… there’s something unique that happens in social settings maybe, so thinking about how we can include that sort of thing, so not just an ad on the side of the bus. I’m joking about the ad on the bus, no one has bus money, and as someone pointed out we don’t even have busses. But… very few busses. But, also not ignoring that there is something special and there’s a way of communicating, a way of learning in groups interpersonally that’s important to take into consideration, too. That’s really beautiful.

R: And being less afraid to advertise them in Alabama because they like to hide them around here.

Mod: Yeah. Alright, well, this has been so… so helpful. I really appreciate you… I really appreciate you doing this as a Zoom focus group. This is my first Zoom focus group. A couple of your first Zoon focus groups, too, right? So I really appreciate you being patient and I know there’s lots of, you know, crinkling noises and whatnot, but hopefully, you know, we’re able to capture everything on the recording, but, um, yeah, like I said if you think of anything that you want to reach out and share with Victoria I would really appreciate that and, um, I just thank you so much for coming tonight and spending an hour… wow, it’s exactly an hour… good job… spending an hour here and, you know, talking with me about these issues and giving me so many ideas and so many things to look out for. There’s just… there’s no substitute for that, so I really appreciate all of you taking the time to do that. So we will go ahead and break. I’ll stay on for a few minutes if anyone wants to say anything to me as other people are jumping off. But I… if not, then we will just go ahead and break for tonight. And all you all stay safe out there out in the crazy world right now. Bye.

[Several good-byes and stay safe being said to each other]

R: This is like a job thing or like… but if there was anything that you maybe with gender or anything that like want assistant or whatever, I mean I have like a Master’s in teaching, a history degree and a minor in sociology and I’ve been trying to find a job for a while and it’s weird trying to find a job that UAB…

Mod: Yeah, Libby, do you have some ideas for how to do job searches?

R: So, um, I have a contact for you. We just started a gender health clinic at UAB that opened about three weeks ago. I don’t know if you’ve heard about it.

R: I did.

R: But I know several people there. I don’t… you know, I’m the… I’m one of the primary physicians there and right now we don’t have any employment options but I do know several people there who may have a better idea of what is available at UAB in that space, but that’s my main… my main contact. Do you all have anything, Ella or Victoria? Do you know [name redacted], Ella?

R: The one that runs it?

R: Oh no, I was asking Ella if she knew [name redacted].

R: The one that runs the clinic?

R: Yeah.

R: I don’t know her. I Facebooked her when her post came on, but…

R: She may have an idea, she’s very like well-connected, so I can ask her. We’ve had several… several people kind of interested in similar things, um, so I’ll ask her if she knows of anything, and I can get in touch with you if you would like.

Mod: Yeah.

R: Yeah, I’m basically… having anxiety with because I’m trans and I can’t just go be a teacher because this is Alabama, so.

R: Unfortunately that is the state we live in. But there’s… you may be, um, you know, there are more and more opportunities. It’s tough right now with Covid, but I’m gonna see if [name redacted] knows anything and I can let you know.

R: Yeah, that would be cool.0

R: Awesome.

R: Well, thank you so much.

R: Thank you.

R: Bye… bye, everyone. I’m gonna end. I’ve recorded everything, so I’m gonna end and make sure everything recorded okay. So, yeah, this was great. Should I email the person who didn’t make it and ask if they wanna make the next one?

R: Yeah, that would be great.

Mod: Was there only one person missing?

R: Uh-hum.

Mod: That’s the best showing ever?

R: Really? I thought it was normal for 40% of people not to show up. Yeah, Victoria’s a rock star.

R: I wonder if this Zoom thing… I mean think of the barriers that this cuts down.

R: Yeah. I mean there are barriers with it, like if people don’t have Internet but most people I feel like could find a space where they could have reliable access and feel safe. I mean I would assume, you know… if you were contacted and you say you’re willing to do it the barriers to like getting on a Zoom call are much less than driving to a place and like walking in and all that stuff.

R: Yeah.

R: Or public transportation.

R: Yeah, yeah. No, I mean it’s just… yeah, okay.

R: So after this I’m going to send this recording to the transcription group and, um, I guess I’ll send a follow-up email tomorrow to the whole team and just let them know that it went well and we’re gonna start working on organizing the next one.

R: Okay

[END OF TRANSCRIPTION]
