# Supplementary material for: “It’s behaviors, not identity”: Attitudes and beliefs related to HIV risk and pre-exposure prophylaxis among transgender women in the Southeastern United States
Source: PLoS One. 2022 Jan 27;17(1):e0262205. doi: 10.1371/journal.pone.0262205 (PMC8794203; doi:10.1371/journal.pone.0262205)
Supplement: S2 File — (DOCX) [file pone.0262205.s002.docx]

Van Gerwen

HIV Study

File Name: File 2-Audio Only

Length of File: 70:30

Mod: … this focus group. Has anyone here ever been in a focus group before? (name), are you nodding yes/

R: Yeah.

Mod: Okay, so a focus group is really just a very sort of intentional conversation. And you’ll see I have… here’s my list… I have a whole list of things that I had picked out for us to talk about, but that’s just a preliminary list. What I’m really more interested in is hearing your thoughts on these things. So you’ll see from my questions kind of the ideas that I’m trying to get at but if I’m not asking the right questions, if I’m not, you know, getting at the things you think are important for us to know, then I want you to say that and I want you to tell me the things that you think that we need to know because this is really about hearing your thoughts on these issues. We can’t, you know… we can’t do the kind of work that we’re trying to do, we can’t help in the ways that we want to be able to help if we’re not hearing from people and hearing their thoughts, their opinions. The other thing I want to be really clear on is, you know, of course, you all signed the participation forms and so you know that everything that we’re gonna say here tonight is confidential. But there’s another level of confidentiality in a focus group and that’s this… I’m gonna ask what does your community, what do people you know think about, and so you’re free to say, well, my opinion is this and my experience is this and I did this once, and these sorts of things, but you’re also free to say, well, I’ve heard other people say that they think about things about this way. And what’s really neat about that is that instead of just me having a one on one conversation with each of you, we’re able to bring in lots of different thoughts and ideas. So if you’ve heard people say certain things that you think are important for us to know throw that in there. I’m not just looking for, you know, what is your own personal opinion on these things, but tell me all the things that you hear when you’re out and about, when you’re on social media, when you’re talking with other people. Because that way we’re able to collect all sorts of different viewpoints and have lots and lots of information to start building on rather than just the perspectives of a few people. So you’re kind of acting as reporters in a sense, and that’s encouraged here, that’s what we’re looking for, so feel free to do that. From time to time I might stop and say, you know, I haven’t heard from [name redacted] on this topic; can you tell me what you think. But please don’t ever feel put on the spot. If you don’t wanna talk at a certain point, if you don’t have something that you wanna say just say, well, yeah, I’m still thinking about, or say, no, I don’t have anything to add. That’s absolutely fine. You know, I find that once we get started talking it’s just like we’re having a conversation and there’s, you know, very little risk of us not having enough to talk about. But if we do, you know, start to slow things down I might ask for people to chip in but please don’t feel put on the spot by that. So before we get started are there any questions that you wanna ask me about what we’re doing here and what we’re trying to accomplish? Alright. And, uh, Dr. Vanger, when have we heard if other people are going to be joining us? Do I need to pause for a moment?

Mod2: [name redacted] has reached out to the rest of the participants, but we can continue to do introductions while we wait and see if anybody’s gonna join us a little later. So, [name redacted], would you like to go next?

R: Uh, I’m [name redacted], I’m 47… um, I transitioned four years ago, and I am in a committed relationship with another girl. Um, and, um, I mostly attracted to women, I consider myself lesbian, and that’s the way it’s been my whole life in the sense that I always felt like a girl that wanted to be with girls, in the girl role. So basically that’s it.

Mod: Great. Welcome, [name redacted]. [name redacted], do you want to introduce yourself?

R: Yes, Hello, my name is [name redacted]. I’m a proud black trans woman and I reside in the state of Birmingham, Alabama. I am currently the president of the Community Advisory Board overseen by Libby, so I already know Libby, that’s how I met her. And I am just an avid advocate for black trans women. I work in public health and HIV and Equity and I do… I work with HIV care and I work with UAB as a standardized patient, who with (names doctors, hard to catch names for spelling), so on and so forth. So I’m an avid advocate and activist in my community for black trans women and I’m just excited to be here opening the conversation, engage in the conversation. I hope to give my viewpoint on a lot and I hope to learn a lot, as well. So thank you, everyone.

Mod: Excellent. Welcome, [name redacted]. [name redacted], would you like to introduce yourself?

R: Hey. My name is [name redacted]. I am the project recruiter case manager and acting coordinator right now for the (sounds like T-Hill) program at BAU. My pronouns are she or her. I have been active in the community for several years, former president of the (06:04-inaudible), so, yeah, I’m excited to be here. Thanks.

Mod: Alright. Fantastic. So we’ve got a lot of diverse backgrounds and that’s fantastic, so we’re gonna hear from all different parts of the community. This is gonna be really exciting, so are you guys ready to get started? Ready for my big list of questions?

Mod2: Before you start I just wanna let everyone know we have a couple members of the research team on the call, too, that we won’t be necessarily interjecting but Victoria, Andres, our two medical students who are working on this project with us and then Letitia (last name) is one of our co-investigators, so I just wanted to make sure everybody knew who those folks were. But we’re gonna be non-video and muted, so everybody enjoy. We’ll talk at the end, as well.

Mod: Okay. Alright, so let’s get started. I can already tell that we’re gonna have lots of opinions, lots of things to hear about for this. So my first big question is tell me about HIV in your community, among people that you know. What are people’s thoughts currently? Do people talk about HIV? Does this seem to be a major issue? Go. I know that’s a big question.

R: Okay, I’ll start. Um, so I know for me, within my community HIV has plagued a lot of my community members as well as family members and friends and it’s kind of like a silent killer. It’s not really talked about as much as it should because of stigma surrounding people, you know, that live with it or people that work within the HIV care field, they associate people to either having HIV or, um, contracting it from someone that they closely work with or, um, you know… so a lot of people still have stigma surrounded around, like, you know, you can die from it. Like it’s this big black plague that no one wants to talk about, like it’s so deadly, that oh my god, if I look at you I’m gonna die. So, you know, it becomes a conversation around how do we dismantle stigma around HIV while ending the HIV epidemic together as a collective. So, um, it’s not really talked about as much as I would like it to be talked about. Many people think… still have this big bubble of stigma surrounded around HIV that, you know, is just like kinda like the unforeseen death that everyone knows is there but, you know, we just want… we just won’t talk about it.

Mod: Sorry, I went on mute. So tell me about other… other perspectives. Do people in your community, other trans women that you know, do they talk a lot about HIV as a big topic of discussion or no?

R: Hey, so in my field… well, previously, before I started working here there was no talk in the trans community as HIV. Um, no one targeted HIV women, or trans women about HIV, no one told us anything about it. It was much like [name redacted] said, it was very silent towards… it was not directed… it was always directed at gay men, and never the trans community. So, yeah, it was just something that had I not already previously known about it, it would, you know… if I was a teenager, you know, it would have been something that would never have been mentioned as a young trans women that, hey, this is real, but…

Mod: Yeah.

R: And even in today’s commercials for PrEP products or HIV commercials they just started adding trans women and then if you really look at it some of ‘em are just drag queens instead of actual trans women.

Mod: Wow. Man, you got deep into my list of questions. (laughs) I can tell we’re going to have a lot to talk about there with… with ads. [name redacted], what… tell me about your experiences. Do you hear a lot of people talk about HIV? Is it a concern?

R: Well, in a way, yes, in my occupation, I’m a cancer doctor, so I have patients who have HIV and AIDS and I treat them for, but sometimes my career has been influenced by the HIV history because it started when I was in high school really and my entire years of med school, happening then, even it’s often to keep up with then, and I remember seeing how it was in those times and we still see a little bit of that, especially with (11:28-inaudible) transgender patients. And I agree with (name), my country at that time many of the people that were mislabeled as transgender were actually transvestites or gay people that were in the sex business to survive, whatever the reason was, that who passed themselves as transgender. And, unfortunately that also gave the transgender community an image that didn’t belong to them. It only made their life more difficult. On a personal level, um, I have my godmother’s brother died from HIV, I have a second degree uncle who died from HIV in that time… talking about the time when people disappeared from public life. I also have another second uncle who I believe caught HIV from the one who died by sharing a… a… a shaving blade. So I think I mainly see as a professional level and in a way the personal level. Some things have changed but even up to this day you see patients that remind me of how it used to be thirty-five years ago and that’s very sad. (12:54-inaudible) sad because in what I would define as the upper socio-economic levels people still feel to some degree that that’s something that belongs to minorities; there is really not a full understanding of how democratic this disease is.

Mod: Yeah, yeah. So that’s really interesting, and it sort of touches on some things that both [name redacted] and [name redacted] had said. So from the perspective… you know, thinking specifically about other trans women that you know, who… who do people think is at risk for HIV? Because that’s such an important part of the story of HIV, right? The ways that it developed historically, I love that you pointed to the history of it and our understanding of HIV, you know, historically in the eighties we associated with certain groups and then it started to change. Who now do you think that other people in the trans community… when you say who’s at risk for HIV who would they say?

R: Well, I haven’t specifically asked that question but what I feel is that many people associate that with certain types of sexual practice, okay? I personally think that there is a lot more way, in terms of promiscuity, that’s with people than with the sexual practice itself. We still talk about men who have sex with men and that’s a term that has been coined and has stuck, has stuck there, and it truly does not represent what the risk is. It’s not about gays anymore. It’s about how you get your sexual life and certain practices, that’s all. At least how I understand it, no?

Mod: Okay, okay.

R: But that’s not something that, at least in the groups, or with the people, transgender people I talk to, it is not a topic that comes up very often.

Mod: No? Okay. Great. What do others… what do others think?

R: Well, I feel as though the new target population is indeed trans women, especially black trans women. So, you know, as a black trans woman I am, you know, more objectified and I am more systemically, um, you know, prone to, you know, contracting HIV, you know, or either already having it because of what society has placed on us as trans women, as black trans women for stigma purposes. So, you know, everyone already think that all trans, all black trans women have HIV already. You know, it’s just like a given. And so for some people it’s like, well, I don’t even have HIV, but that is the stigma that is attached to us. We’ve seen it more from MSM, men with men, now to, you know, trans women more of a modern day, um, like I said, stereotype and targeted population.

Mod: Yeah. Well, that’s powerful, and the fact that you used the word targeted. So can you give me a sense of who… who is targeting? Where are you getting these messages that, you know, we’ve sort of shifted away from focusing on MSM and now it’s black trans women that you’re hearing and that you’re feeling are the group that we’re concerned with now for HIV risk? Where… where do you think that’s coming from?

R: Well, it’s coming from the cisgender community for one, it’s coming from multi-straight media, you see more ads now with trans women in HIV commercials. It’s in the media, um, you know, and working in health care, like the numbers are rising, trans women being homeless infecting with HIV and needing more HIV care. The numbers… you know, it’s factual. You can look at the rates and just look from the history of time for men sleeping with men… they used to group us to men, as if we were men, but only, you know, in the last five years or so or maybe last… we’ve been, you know, separated from men with men and now trans women is, like I said, the target population, ‘cause like it’s all over the media, the news outlets, you know, on TV, within our community. You know, people are talking about it like, you know, it’s just there. The numbers and things like, you know, are what people are saying.

I: Okay. Interesting, interesting. So we’re gonna come back to a lot of those same things that you’ve just talked about, about visibility, that sort of thing. [name redacted], do you wanna… do you wanna weigh in here?

R: I think [name redacted] said it best. It’s like the… it’s… the target population is now the trans women, but they’re still looping it in with MSM, so, um, I don’t think I can add anything extra to it.

R: I’d like to add something… Um, it’s sad… something that I’ve seen recurrently during my career as a trainer here and (18:40-inaudible), if I presented a transgender patient, my attending, the first thing the attending will say, you need to get HIV, chlamydia, syphilis, that’s a person… I see the person… because the person is transgender automatically he looks at promiscuity, just (19:07-inaudible). So, sure if the person works in the sex business you make… you have to do it, but just because the person is transgender you don’t. When in med school or in residency you present a case about a transgender person it’s almost invariably because the person has some sexually transmitted disease, not because the patient simply happened to be transgender like some way you can assure her (19:35-inaudible). So there is, in a way this psyche and this sub-consciousness, subconscious of, um, uh, make up professionals, there’s still this bias. And I even asked one time about just presenting, introducing transgender people to the make up without making them, associating them to a particular disease that is nowadays identified as just transgender. And really what I found is there is no interest.

Mod: Interesting. Okay, so you’re saying it’s sort of the pervasive… at least within the medical community there’s just this connection that when we… do you think it’s… do you think that it’s maybe because we… we tend to talk about, and I know that we often train people to think in terms of the LGBT community. Is it that the T gets lumped in with LGB and we know that we’re supposed to be concerned with sexual risk behavior among gay men in particular and so does it sort of like rub off on trans individuals? Like I know I’m supposed to ask something when I see an LGBT patient… oh, yes, it has something to do with sexual risk behavior.

R: Uh-hum.

Mod: Do you think that’s what’s happening?

R: Maybe to some degree, but there is also… if you talk to people, if you could really read their minds there is separation, many… many of them… between us, straight and them, LGBT, no? And that comes with a judgement, a value, more. Nobody talks about it but that reinforces the idea of attaching what is perceived as risky behavior, certain diseases, seen as punishment to that particular group. The problem you suggest of LGBT population may have merit but it’s not because they’re LGBT, because of the socio-economic environment in which they go and unfortunately it’s how we continue to wrap things around the idea of risky behavior equal… LGBT equal risky behavior.

Mod: Yeah, yeah.

R: People look at you as if they came from a different planet and may think twice about grabbing your hand or shaking hands. That’s something that we see a lot.

Mod: Yeah, yeah, that’s par… [name redacted], chime in on that, yeah.

R: Yeah, so I think that the history of HIV and health related, you know, disparages have been so derived in our history as LGBTQ folk that people don’t see us anything else but HIV or sexual health related, you know, commonalities. So, you know, with that being said, people are totally oblivious to the fact that there’s straight people have HIV, too. You know? But I think that people are so conditionally trained that they don’t wanna train their minds to see above, you know, and outside of the box of the ramifications of this is just not an LGBTQ epidemic; this is a world epidemic.

Mod: Yeah.

R: And what people don’t realize is, you know, all people can contract HIV just like all people can contract cancers or, you know, diabetes, etcetera, etcetera. But since the people has compartmentalized us into compartments it has to be in their minds, oh, this is an LGBTQ disease or this is, you know, a gay disease or this is a trans disease. You know what I’m sayin’? Like, hey, we are gonna (23:40-inaudible) in the beginning of the history of time. Just give it to them, so we can take, you know, the focus off of us and it won’t be so bad that, you know, people have projected HIV or whatever, you know, if we can get with them and say, hey, it started with them and it’ll end with them and now we just see in our community where stigma has migrated from men sleeping with men, gay men, down to trans women. (24:12-inaudible)

Mod: Yeah. I… you… hearing you say that it made me think of… I used to, you know, teach classes on HIV and I would talk about the prevalence of HIV in sub-Saharan Africa and students would be… okay, they’d be writing that down, high prevalence there… and there’d always be one student who’d say I didn’t know there were that many gay people in Africa. And I’d say, oh, no, no, no, no… HIV is not only… and then I’d be like, oh, I’ve done a terrible job of teaching these people. I’d have to take several steps back and explain, you know, no, HIV is… can be, you know, contracted by anyone and it’s behaviors, it’s not identity and that kind of stuff. But, yeah, that’s… I absolutely hear what you’re saying about that. Other… other comments about HIV before we move on to our next topic? If you think of anything else around the things that we’ve just talked about even once we move on, if you remember something, say, oh, I remember something I wanted to say about that, just pop it in.

R: I remember being told twenty years ago in my country that 70% of women who had HIV were (25:36-inaudible). It just gives you an idea of… such a… going back to what you just said, this… this perception is so twisted, so pressing up to this day, when you talk to guys, guys still believe that they’re the active ones. They’re fine; it’s the problem of people of who (26:03-inaudible), you know, and it’s amazing, I mean as somebody who grew up surrounded by guys, how many of them have got the idea and how many of them still look for transgender people because they also have their fantasies. It’s all a double standard, and it’s all a game, a double discourse all the time.

Mod: Yeah, there’s so many… so many myths and misperceptions and it all comes back to, you know, what you’ve all been saying about the fact that there’s stigma and we don’t talk openly and we don’t, you know, directly address those misperceptions when we hear them. We just kind of say, oh, yeah, that’s not… that’s not proper to talk about, we shouldn’t… we shouldn’t discuss that. Yeah. Um, so the next kind of big topic that I wanna talk about, and we’ve already touched on it some, so I know we’re gonna have lots to say about this, is talking about PrEP… uh, so Pre-Exposure Prophylaxis… um, you know, there’s several different options available for PrEP now. And you’ve already mentioned seeing, you know, ads and whatnot. Let’s talk a little about PrEP and really focusing on PrEP in the trans women community. Do you hear talk about PrEP? Do people talk about, oh, PrEP is a great idea and we should really look into that, or is there just silence around PrEP? Talk me through some of that. And this is a great… you know, I mentioned earlier… tell me what you hear people say, tell me what other people’s opinions in addition to your own. Talk me through that. What are people saying about PrEP?

R: Hey, can I… so, um, what I hear about PrEP in my field all the time, um, again, that they are still (28:04-inaudible). There was a survey on a PrEP question the other day…

Mod: Uh-oh.

R: Hello? Am I there?

Mod: You still there?

R: I think I got disconnected for a minute. Can you hear me?

Mod: I can hear you now, yeah.

R: Okay, I apologize. So there was a question on a PrEP survey the other day… are you a man who’s having sex with other men. My response is no, but then again, if I’m trying to get PrEP, what is, you know… I have to say yes because clearly I’m a trans woman who has sex with men. So there’s… there’s this stigma that if you try to get on it and then there’s this… I’ve had a pharmacy representative tell me one time that sixty percent of trans people don’t realize that they’re at risk for HIV, that there was no need for PrEP in the past, in which this is a big false, because that’s always been something in the back of mind, you know, am I gonna sleep with someone who’s not protected or, you know, those things. But, yeah, that’s just my own thing.

Mod: Okay, so the idea that the way that PrEP is presented, the way that it’s marketed and even in some cases the way that you would get access to it implies that it’s only for one group of people.

R: Exactly. And then you also have to think about it, there’s always this thought in the back of your head, we’re taking hormones, what are the side effects that it will have when you’re on hormones and the PrEP.

Mod: And do you feel like that’s the kind of thing that the health care providers that you see, that prescribe hormones, do you feel like that’s something they would know the answer to if you were to ask questions about that?

R: My current provider, I do, but prior to this probably not.

Mod: So provider education would be important, you think.

R: Correct.

Mod: Other… other thoughts? Tell me some other views among other trans women that you talk with or that you, you know, interact with on social media… what are the thoughts on PrEP?

R: So, um, PrEP is kind of like, you know, another silent thing that isn’t talked about, it’s just like, you know, it isn’t there so a lot of people don’t even know what PrEP. So, you know, we don’t really see a lot of advertisement surrounded around PrEP for trans women, only gay men. And I guess, you know, stating that stigma again, it rolls like back around to them grouping us in with gay men. And so, you know, the health care providers aren’t talking about PrEP, they aren’t recommending PrEP. You know, it’s kind of just like… I think it’s just like, well, you know, they are the target population and we gotta keep our numbers up, so, you know, if nobody’s contracting HIV within the trans community how do we get our numbers, how do we get paid. So it’s kind of like a big money, you know, gamble thing, I think, so it’s just not talked about and there aren’t many resources that, you know, trans women can go and be afforded to… to even afford PrEP because lots of trans women, you know, especially trans women of color don’t have health care and can’t afford health care, so how do you even talk about PrEP when you don’t even have health care. You see what I’m sayin’? So it’s just like, you know, that’s, you know, undisclosed thing that there aren’t many resources around it and, you know, people just can’t get it.

Mod: Hmm, okay, so it’s an access issue. So I’m hearing it comes from a lot of different… like people aren’t told about it, people don’t know what affect it might have and they can’t access it, so there’s all sorts of different things working against the use of PrEP among women, among trans women.

R: Yeah.

Mod: Okay. [name redacted], what… did you have…

R: Um, a couple points. One is, um, forums in which I have participated or Facebook groups, never comes up. It’s interesting, and I wonder if it’s… maybe it’s because, well, a percentage of girls like me, we date girls, you know?

Mod: Yeah.

R: Some, especially, I guess all the ones look for more committed relationships and also because even those who date guys, they don’t see themselves in a gay relationship. They see themselves in a sexual relationship and normally sex… sexual couples don’t discuss PrEP. It’s just not something you hear your friends talk about. But why? Because we are not… we are people… I feel like a woman so I relate to my partner as a woman, so my partner sees me as one, so sexual couples, he’s a guy, why would they think about PrEP more often than a non-trans sexual couple, you know? The other thing about PrEP is, um, if somebody comes to me and talks to me about PrEP after hearing that I’m in a committed relationship I would slap them in the face. Why? Because it’s not about me being trans, it’s not even being about… whether or not I anal sex or not, it’s about whether or not I’m in a promiscuous type of sexual practice, okay? And that’s just my personal take on it, that we… if you are in a committed relationship there’s no point really doing PrEP as long as really committed. Yeah? But you could say, well, what if the person cheated. Well, that’s true for all sexual couples, no?

Mod: Yeah. That’s… you’re blowing my mind here. I’ve written down several of the things that you just said

R: One more thing. There is a good number of transgender girls who have had surgery, you know. Sure, not everybody can have surgery and not everybody wants surgery, but those who have had surgery can engage in what they will completely normal woman to guy relationships for the simple reason they don’t see that as a high risk, um, uh, practice. (35:12-inaudible) want to date guys and they will go to bars to find somebody to go out with, but believe it or not it never comes up. You can talk about sex, but PrEP, that’s not come up.

Mod: That’s really…

R: (35:29-inaudible) because PrEP has… just the fact that you have to prevent something means that you could get HIV cannot… it means that you’re doing something wrong.

Mod: Yeah. I gotta write that down. Wow. Uh, [name redacted] and [name redacted], what are you… what’s your reaction to hearing those thoughts on why, you know, this idea that PrEP is for certain types of people who are having certain types of maybe promiscuous relationships, but that’s not me, so why would… why would I even be thinking about PrEP? Why would that even be a part of the conversation in my relationships? Any… any…

R: Yeah, you know, she hit it definitely on the head. Like I was sayin’, goin’ back to compartmentalizing stigma, you know. Cisgender couples feel as though this is a same gender loving relationship, sexual relationship thing, you know… that it doesn’t apply to me. I’m not messing with men, or, you know, women sleeping with women, or whatever the case may be, that doesn’t apply to me so they’re just like grouping themselves apart, you know, from us. I just totally agree, you know, (36:53-inaudible).

Mod: Yeah, yeah. Wow. Um, so other thoughts about that… that, sort of that why would we talk about PrEP, given the relationships that we’re having, yeah. Any other concluding thoughts before we move on to my last question.

R: If you…

Mod: Yeah, sure…

R: If you look at these commercials they always refer to our lives as risky behaviors, why do we have to be referred to as risky behaviors? That’s so… I mean it’s just… it’s crazy. But everything else that they said I agree with 100%, other than my life is not a risky behavior and none of my trans sisters are risky behavior, either, so… yeah.

R: I agree.

Mod: Wow.

R: You see the advertising for gay guys sometimes and you see too often, too, totally normal guys who are just hooking up together, and maybe they’re just dating. My best friend from school is gay and he’s been with his partner with ten years. Why do they need to be associated with PrEP? And like what’s the point in advertising PrEP to that kind of people and we really… yeah, sure you have to look at gay people, transgender people, but also there are sexual (38:19-inaudible) gender… you have to look at everybody because it’s really this… the behavior and when the behavior is (38:28-inaudible) sexual practice, but when you’re in a committed relationship…

Mod: That shouldn’t… yeah.

R: Right.

Mod: By definition it shouldn’t be an issue.

R: It shouldn’t be an issue except no matter what your label, color…

Mod: Right.

R: You know… (38:50-inaudible) whatever is.

Mod: So… so my last big question was going to be, well, how could we effectively market or, you know, inform… you know, thinking about educational programming or, you know, pamphlets or fliers or anything, how could we talk with trans women about PrEP? But now it doesn’t even seem like that’s the right question, right? So earlier on… I can’t remember who mentioned it… I think it was [name redacted], mentioned something about the ads aren’t right, they say it’s trans women but I can tell it’s actually just a, you know, a drag queen and that’s different. And so it’s not even a, you know… it’s not even a question of representation or of seeing yourself. You’re saying like trans women as a group, as a specific identity, that’s… that’s not even a group that we need to be thinking about marketing PrEP to. We need to be thinking… [name redacted], you were saying it’s about particular behaviors that people engage in, it’s about particular types of relationships that people engage in, so talk me through that a little bit. What would that… is it… is it important or meaningful to see trans women’s lives represented in that or that not… does that not even matter? Is that not an issue? Thinking about, I mean, you know, I think you’ve all talked about seeing particular, you know, ads on TV, for instance for PrEP, but it… yeah, oh, I’m just… talk… talk me through that. I’m… I’m… you’ve blown my mind here, so talk me back down.

R: Um, yeah, so for me I think that, you know, people, you know, want to see representation of themselves. So, you know, if you put a trans woman or black trans woman on the (inaudible) or PrEP commercials trans women will feel more prone to believe, you know, that it’s applicable for us. I think that only recently they had one PrEP commercial with Hayley Sahara from Pose. I think that just was a recent commercial. As a trans woman, she was depicted in a PrEP commercial and that’s the only one I ever seen. So only now are we beginning, you know, just splashing the surface of being able to create representation centered around trans women, you know, for PrEP and HIV, and not just grouping us in with men sleeping with men or gay men. So I think what it’s gonna take is like I said, making PrEP affordable, and not just, you know, leave it out there saying, hey, this is PrEP and (42:04-inaudible). No, you have to meet you with where they are, realizing that everybody can’t afford health care insurance and everybody can’t afford medications, especially everybody cannot afford PrEP. So how do we dismantle the system of again of affordable health care access for (42:25-inaudible).

Mod: (inaudible) (laughing)

R: … so that our people know as trans women that we are represented. Of course, you know, HIV affects us. Of course it plagues our community. Of course we are a part of it. I’m not negating that, but you have to give us some legroom and some room to stand in our own existence; at least give us our own existence in the doggone health disparity. Come on now, we can’t even get our own health disparity, grouping us with gay men… come on! So what we have decided about having a conversation centered around getting people within the community that are willing to speak out and be a face of representation for PrEP or HIV and talking to them to see exactly what trans women need, you know, in our respective communities, and that is only when things will begin to change. You know, we are the experts. We’re not the only expert, we are the experts so come to the experts. You know, use us for these things so that we can communicate around prevention, around HIV care. If a lot of, you know, trans women seen that a trans woman was the face of HIV prevention or even PrEP prevention, whichever the case is there are a lot of trans women would be prone to say, okay, this can happen to me and, you know, I am a part of this community, let me listen and take precautionary measures or, you know, if I am, you know, infected let me go and take care of myself. But if you don’t see no one who looks like you how can you take the affordable measures and the precautionary measures to do anything regarding your health? So it’s just a representation thing for me.

Mod: Yeah, representation. I am curious… so you mentioned, you know, actual… I mean access to PrEP as a potential barrier just in terms of the cost. Are there other barriers? Thinking about, you know, if we had good trans women representation what would the other… what would be other potential barriers that we would need to think about? What are other things that keep people from even thinking about PrEP in addition to, obviously, access, in addition to thinking that… and possibly, you know, correctly so that they’re not at risk or that it’s not something that they need to be concerned about given the lives that they’re living, but what else? What are other barriers, just generally that people think about?

R: Um, uh, I would say stigma would be a big… a big one, you know. If people associate you with, you know, any type of medications, PrEP, whatever it is, oh, you automatically, you know have HIV or some kind of, um, you know, infectious disease. So I think that just having people speak out against stigma, saying that, you know, hey, whether I am HIV positive or not these are the things that can happen to you and me, as well, so how do we as a collective, you know, build and create change. And, you know, you have to think about transportation. A lot of people don’t have transportation to get health care, you know, and so, um, how can we come together to some kind of conclusion to even get PrEP to people or get people to PrEP. You know, how can we interchangeably work with gay men in order to bring more representation to the trans community as far as, you know, trans women, you know, being on PrEP, work in these PrEP clinics, you know, getting PrEP for free or affordable price and, you know, different things like that. Just give us a seat at the table to represent us in any avenue, and I think that, you know, there’s a lack of representation, as well. We’re not represented as a trans women community because gay men are doing it for us. A lot of these community-based organizations and in these health care fields trans women are not on the front lines representing us or ours. They either have gay men doing it or cisgender women. And, you know, I’m not taking anything away from gay men or cisgender women, but it’s women, trans women in the community doing the work. If you try hard enough to find these trans women to let them be on the front line a lot of trans women, you know, will be saved and prevented from having and contracting HIV. You know, a lot of more trans women will be on PrEP, but as long as you have gay men working in place of them and cisgender women they’ll not be any applicable change because we are not represented in the health care field. We’re not! So trans women don’t see trans women represented in the health care field so they’re not gonna be prone to be able to be on top of their health and talk about and have active conversations surrounded around preventive measures and taking care of yourself. It’s never gonna happen until people let us represent us and hire us to work these jobs and do these things and represent us, and that’s what I have to say about that.

Mod: (laughs) And I love that you’ve… you’ve pushed here what we mean by representation, so it’s not just, okay, you know, we have media representation or we have representation on social media in different educational things, but you’re talking about, no, I mean actual, being a part of health care providers, working front desk, being there… like that’s… yes… yes… that… yeah, that is… that is a critical, sort of pushing beyond what we normally mean by representation. It’s not just who we see, but it’s who’s actually there, who’s actually involved. I wrote down you said ‘give us a seat at the table’ and I underlined it and that’s exactly… yeah, that’s powerful. Other… other thoughts on that, on what we mean by representation, on potential barriers to PrEP use? Tell me more. [name redacted], you…

R: Well, it’s a complex problem and I think that one hand you have to reach everybody, on the other hand you have to reach certain groups, but you don’t want to leave out anybody but you don’t want to stigmatize any… so it seems part of the solution has to be on like a tier solution. One hand, (inaudible) PrEP in general should be for everybody regardless of who you are, but not necessarily advertise it (49:42-inaudible) or to any particular group, unless you do it as part of a combined that has all sort of people, you know. Um, but on the other hand and going back to what (inaudible) said it’s very important that (49:58-inaudible) groups and their concerns are addressed.

Mod: Yeah.

R: You may not be able to address the concerns of the trans community, represent… what… point-five percent of population… until you stop the bias. But you can certainly go and look for the community, try to find the people that can help you navigate that community and reach them. That’s very, very important. We see community… it’s diversity, because it’s very… I always said that trans communities or micro cosmos of society. We all have in common, we’re trans… you remove the trans part and we’re as diverse or different as everybody else. Then the other fear in relation… well, you realize are group oriented approach and then general approach inside each and everybody regardless of who you are, what your gender status is, sex is, attraction is, it’s about something that you do for, um, um… yeah, to prevent infections when associated with certain practices. The first thing is you have to teach health care professionals, not the way we are teaching them now, which is disease or person with these characteristics equal this way, equal this intervention. You have to teach them that you’re dealing… they’re dealing with people. You have to teach them to leave their biases behind, because I see that every day. The reason I don’t (51:36-inaudible), they know who I am, but I know I’m in that position… I know I’m in a position (51:45-inaudible) but I can see that in their eyes and I’ve seen them do that to other people. So you have to teach them how to approach people, how to do it not from a judgmental position, which you are the doctor, but be nice enough to offer that person something for that, you know, really bad behavior but just simply offer something to somebody who engages in the practice regardless whether or not they’re sexual trans, whatever. Yeah? And the other thing is how you talk, not just how you feel, how you sell these. You don’t want to sell in a way that the person who’s trying to sell these to is going to stigmatize them more. You don’t wanna sell to a trans person because you sell your body or you have sex with too many people, especially because I don’t think many trans women just want to have sex with many people, you know. And maybe some gay guys, but most trans girls, if they do it they do it because they mean to, society doesn’t give them another option. So how do you remove all those stigmas to deliver a clear message? And a clear message that is not equal trans, a clear message that is equal, risky behavior without any judgment, no judgment. You should not judge them here, you should not attach it to anything else, and just provide something because we are empathetic, we understand what you’re going through and we want to protect you, whether you do it out of love or out of need or out of you just want to try, you know, (53:40-inaudible) Because PrEP could be something that you could offer to a woman who has a husband that cheats on her every day, you know.

R: And I have something else to say, Erica. I meant to say change the verbiage on these applications, on these PrEP surveys, include trans women. Another thing that withheld trans women from being on PrEP or anything like that is the verbiage, like [name redacted] mentioned earlier. No trans woman wants to be reminded every single time that she does a application or a survey, that, you know, she was born as a male. We already know that, that’s a given. You know, give us a chance to exist, a place… are you man sleeping with man or a trans women, male to female or trans man, female to male, you know. Let us be able to exist within society and so that’ll be more prone and it’ll be more opening for us to be joyful about, you know, joining PrEP, hey, they’ve included us on the survey, you know. The question is am I trans woman… of course. That’s me. That fits within my ramifications. That fits within my rights. I’m a trans woman willing to be on PrEP. If I only see am I a male or female or gay, where is the trans women… no, I don’t care about PrEP, I don’t even wanna be on PrEP because they don’t even include me in it. You see what I’m saying? Trans are audience, hold them accountable, don’t dis-include us. Every time you see a survey’s placed out and you read it without representing trans women as a trans woman is not an option delete it, throw it away, redo it. Trans women is in everything and we need to be included in everything as well just as well as everybody else. Put us on these surveys and these applications so that we’ll feel more prone to believe that we are cared about and that our lives matter and it’s not just a life of risqué, you know, kind of behavior. You know, we’re just not at risk like [name redacted] said, you know, we’re included and we can take preventative measures to do whatever it is that we need to do. Include us, put us on these surveys and applications.

Mod: [After some dead space] Who keeps (inaudible) me? Alright, no one touch. Alright, I’m not gonna touch the computer. I love that you said that, and we’ve been… you know, we’ve been telling health care providers for years, you know, it seems like such a simple thing, update your forms, do it now, and that is just… it is so meaningful. And like you said, it makes people feel seen and that is critical. You are never gonna get past that if you don’t show people that you’re seeing that they’re valued and welcomed and cared about, yeah. So, ugh… yeah?

R: (57:07-inaudible) comments, please. It’s very important to have access. If it were for me, PrEP should be available like the next day pill, yeah? You should just be able to go to the pharmacy and buy it at cost price, or even have access to coupons and just get it because it’s important. You shouldn’t require to see a doctor for something so vital, you know. Um, you know, because the doctor and the doctor’s fee and the co-pay become another barrier. That’s real. And then it’s important to engage the industry, the industry, not just the pharmaceutical industry, but the employers, like institutions. Why? Because they need to make all this part of their education, diversity education. You need to change the language, yeah. How would you present it? How would you describe who is transgender? You know, try to remove the sexual part from root, yeah. Look, I’m an attending at UAB. The transition (58:20-inaudible)… the policy that had just been published by the VA, the national VA guidelines, so they could adapt them because in 2017 (58:33-inaudible) how to deal with transgender people. And I’m not (inaudible) UAB, it’s just very widespread, it’s quite common, everything we see on TV for most people it’s in a different universe.

Mod: Yeah, yeah, that’s so true.

R: You look for the… look for an LGBT community at UAB or at Children’s. You have to find it discreetly. Why? Because we don’t want to upset people. How can you really play your role… and again I’m not talking about UAB, just in general, this is every institution… if we do not come out openly and transparently we cannot have secret clinics. (59:34-inaudible) need to treat HIV people. I know it’s no longer just that, but what’s that… what’s that that people understand? So we need to change that; otherwise we continue to reinforce certain ideas and unfortunately prejudice.

Mod: Yeah, yeah… absolutely. Um, so other… other thoughts about that? That was kind of my last big question, but we’ve talked about so many critical things here… I mean you guys have just been… you’ve provided so much information that is so useful, and things that we haven’t heard before and perspectives and so this has just been… just so… so powerful. Are there other things… I said before if I don’t ask the right questions tell me and tell me the answers to those questions. Are there other things that we need to know, other questions that I should have asked? Tell me that.

R: I think that it would be interesting (1:00:55-inaudible) if you could have the same focus group with people who are not trans, who are just (inaudible), okay? Who are cisgender… see what they say, because they will tell you the other side of the coin.

Mod: That would be so fascinating. It would be a very short focus group because they’d just say it doesn’t apply to me.

R: Well, some people will say that but others will probably surprise you. I mean what I’m trying to do is not assume any kind of response from them.

Mod: Yeah.

R: But you will be surprised because you could question that that doesn’t apply to me and you will see a background of race or discrimination or interest or god knows what, but that is something that we have to address at some time because that’s in the way of the root of all the other problems you see, that’s why trans girls don’t trust other people, because of that. That’s why they feel that. That’s why they feel that the system tries to corner them, label them, because of those things. You start with solution, yes, don’t find it in us… I believe it’s also outside, you just have to look different.

Mod: That’s a really good way of thinking about it. [name redacted], what do you think?

R: Um, I wasn’t gonna speak on that, I was just gonna speak on another point that I came up with. Another point is these health care officials and providers and just people within the community period need to stop thinking just because, you know, a girl is HIV-positive that she can’t be an actual representation of PrEP, you know, or getting girls drawn to PrEP. The best expert is the one who has been through it. Allow her to shine through and give her story and her testimony and she’ll be the one to bridge the gap between health care provider and, you know, the trans community by giving a personal account of her story telling them that, hey, you know, I wish there was PrEP, I wish there was access to health care, I wish I had the resources that you have, but now that you have it use it to the best of your ability. You have preventative measures out here. You know what I’m sayin’? Things I didn’t have or things that weren’t prevalent as I, you know, had them. Don’t turn anyone away just because, you know, you’re HIV-positive… you can still speak to the masses the most. I feel as though you can reach a plethora of trans women by giving your personal account.

Mod: Yeah.

R: Because they just wanna hear about how it is and, you know, how you navigated through it and what do you think, you know, a good preventative measure is as a woman living with HIV. You know, give her that chance to speak and to advocate for those who can’t advocate for themselves.

Mod: That would be really powerful. I love that, I love that idea. [name redacted], do you have any… do you have any comments?

R: Also, [name redacted]… everything that I would have said [name redacted] said right off the bat when she first started talking about it… representation, breaking the stigma, affordable health care. [name redacted] hit it all. And that is something that I think she and I see every day as we work in this field, so [name redacted] literally hit every point and she continues to hit every point, so there’s nothing more than I can do or I can say, that working in the field [name redacted] has already said it all. These are the things we see every day with the people that we serve.

Mod: So are there any other… now I’m mindful of time, are there any other big… big things that you want all of us to hear? Are there… are there questions left unasked? Are there… are there big ideas that we need to follow up on or does someone wanna make a grand, concluding statement?

R: I do. I would like to… I’ve been called… I would just like to say we can sit here on this phone call, on this Zoom call, whatever, because of Covid-19 we’re not really in person, and talk about preventative measures and talk about how we can make active change and make it applicable for the trans community. You know, one thing is to talk about it, but like my mom always said, let’s be about change, don’t talk about it, be about it. Actually when we get off this call, call up some of these, you know, trans women or us on the call, and, you know, talk about, hey, how can we get some boots on the ground and get it movin’, because we can talk about it all day long… and all day long, but just talkin’ about it it doesn’t create effective change. You see what I’m sayin’? We only just… it’s talked about. In order to have effective change we have to move and we have to move quickly because people’s… trans women’s lives are depending on this. Every second or every minute that we waste, you know, just talking about, well, okay, we can do this, have conversation around this… that’s not good enough. Conversation is good but let’s put some action plans behind our conversations and actually do what we talked about tonight and actually hire the trans women to go out in these trans women to go out in these communities and speak against stigma and speak against, you know, people not being ashamed to take care of themselves and get on PrEP and speak against us not being included in PrEP, everything that we was discussing on this call today, let’s get some action steps behind it and actually do it because I’m ready… I’ve been ready. So let’s do it, let’s be the change that we want to see, let’s create the change that we want to see. Absolutely, let’s usher in the change that we want to see, let’s do it.

Mod: I wrote that… be the change… I love it. I can’t… I can’t even… there’s nothing more I can say. (laughs) What a great… what a great way to wrap up. Are there any other concluding thoughts that people wanna share?

R: I think you should be able… I mean you should be willing always to upset people, to cross those boundaries, because if you just work on the function based on the comfort zone of those who don’t want to change we’re condemning those who we want to help.

Mod: Yes. Yeah. Any other… [name redacted], concluding thoughts?

R: Be okay with uncomfortable conversations. That is going to be one of the key things. When a trans woman tells you something has offended her, listen. You know, when a trans woman says this is not how it should be about our community, listen, just be okay to open conversations and be okay if you’re offended.

Mod: Yeah, and hear that and believe it. That’s… I mean I think that’s one of the biggest mistakes people make, saying, no, no, that’s not what I meant, I didn’t really offend you. Yeah, you have to… you have to be willing to engage that. Wow. Alright. I don’t know about you guys but I am… wow, this has been an emotional rollercoaster. I’ve heard so much important stuff, and this is just… I just… I can’t thank you enough for sharing all of these thoughts and being willing to talk with us, you know, in this kind of strange Zoom focus group… you know… this is a weird… it’s a weird way of talking, but I feel like this has been so effective and I’ve learned so much and I’m sure the team has learned so much. And I just, you know… this is… this is really important work and I appreciate… I mean all three of you are already doing this critical work in different ways and being a part of this conversation and I just thank you so much for taking this time to talk with us tonight.

R: Thank you for having me. Call me. I’m ready to work, I’m ready to bridge the gap with some ladies and some women is all I can say. But thank you, this has been great. I’m really looking forward to working with you all in the near future.

Mod: Fantastic.

Mod2: I’m going to put my email address in the chat box. If anyone has anything they think of later on that you wanna mail the team about what we talked about tonight… I think most of you have my contact information, but shoot me an email if you think of anything. And like Dr. Austin said, this has just been wonderful and the whole goal of doing research like this when we do focus groups and things is to find ways to put y’all’s insights into action, so what [name redacted] said about doing the work that’s what we wanna do, so we appreciate everybody’s input and I hope you all have a good night.

Mod: Yeah, thank you ladies so much.

[Everyone saying thank you, then recording ends]
