# Supplementary material for: “It’s behaviors, not identity”: Attitudes and beliefs related to HIV risk and pre-exposure prophylaxis among transgender women in the Southeastern United States
Source: PLoS One. 2022 Jan 27;17(1):e0262205. doi: 10.1371/journal.pone.0262205 (PMC8794203; doi:10.1371/journal.pone.0262205)
Supplement: S3 File — (DOCX) [file pone.0262205.s003.docx]

Van Gerwen

HIV Study

File Name: File 3-Audio Only

Length of File: 54:01

Mod: Dr. Van Gerwen has already talked a little about what this study is about, but really the whole point of this study is you, it’s hearing your perspective, your experiences, your stories. So I have a whole list of questions. Looks like a lot, it’s not actually that many questions. But I have questions, but I’m going to ask you to tell me are these the right questions, is that what we need to be talking about once we get into it and you kind of hear the sorts of things that we’re interested in learning about. We’re really, you know, trying to hear from your perspective on these different issues that we’re going to be talking about. So if I ask a question… no, no, that’s not the right question; here’s what you need to know. Please go ahead and let me know that because I’m really counting on hearing from all of you guys tonight? Does that make sense? Okay. So we’re going to be talking tonight about community, so even though I’m going to be asking about your personal experiences I’m also open to hearing sort of your perspective on what other people in your community think about these issues, because then that way we can get lots of different people’s voices in here. Right now we only have three people on the phone, but I’m counting on you to represent and to tell me about different people’s perspectives. So you might say, well, I think this way but I know when I’ve talked to other women in my community they say this, and so I think that this is an important thing to be heard, as well. So I’m going to be asking really for you to reflect on what you hear lots of other people saying as well, people in the trans community here in Birmingham because that’s what we’re really focusing on. So the topic, the main thing that I want to talk about tonight is your thoughts and experiences and feelings about HIV. And the first thing I’m curious to know is tell me about HIV right now. What are people talking about HIV? What do people think about HIV? Is it still a concern or is it kind of passed? Tell me what you’re hearing. What do you think? Who wants to start?

R: Okay, I’ll start. I don’t think it’s as much like it… when it came out like twenty years ago it was kinda like, oh, it’s a secret, it’s a secret… everybody you know… you know, people that were causing it, it was like a really tight knit secret. But I don’t think it’s so much as a secret now, people are, you know, being open and sharing their status, they’re not ashamed to share their status, they’re comfortable with sharing their status with people, you know, whether it’s, you know, someone that they’re intimate with or, you know, just in a meeting, they’re just comfortable with sharing their status. I don’t think so much stigma is associated with it. And then on the other hand, I think the heterosexual community think all trans women are HIV-positive, so, yeah, that’s my starter point, I guess.

Mod: Okay. Yeah, well, we’re definitely going to follow up on that because that’s the kind of thing I want to hear more about. Okay, so here’s… oh, sorry… who’s talking… [name redacted]?

R: Yeah. To piggyback off of what she said that is very true. Um, because I just moved to Birmingham so I spent four years of my college career in Montgomery, so me coming here was a culture shock because people… Birmingham just does things differently, period. (laughs) So when I moved here it was, I guess, a shock or two, men who wanted to talk to me or the heterosexual community who engaged in conversation with me when they found out that A) I was negative and B) that I was (inaudible). It was just a shocker and it was like ‘really’! And it was really even more shocking that they were surprised that I had a college education, just because as a trans woman can’t go to college and obtain a degree, so it was just dumbfounding to me.

Mod: So, sort of putting together what both of you have said then it sounds like there’s sort of a perception, and you’re saying that it’s primarily around heterosexuals, there’s a perception that trans women have a particular experience with HIV.

R: And not even just the heterosexual community. I would take it another step further and say the gay community portrays trans women like that.

Mod: Wow. So like where… are you hearing that at different… like different community centers or out in the community with friends or…

R: I have a really good… I have a really good friend that works with AIDS Alabama and this… I go visit him sometimes at work and it’s just real shocking like when people ask him it’s like, oh, well, where do you work and I tell them I work for (inaudible) it’s like… it’s like an intimidation for them. And I don’t come off intimidating but it’s like, oh, really, and so I guess it’s not the normal and it’s sad to say but that has kind of been the narrative of trans women for a couple years now. But it’s like it’s so shocking that when they don’t meet a trans woman who (inaudible) and since I’ve transitioned I’ve never come in contact with a trans woman that does sex work and I’m just going to throw that out there, so it was culture shock when I met my first trans person… woman… who was a sex worker and it was I wasn’t judging her but I was interested to know like how does that work. But it’s definitely a culture to me, a trans woman who doesn’t match that narrative or preconceived notion that they have of us.

Mod: Okay. And do you feel like that’s… you said Birmingham’s… Birmingham’s different. Do you feel like that’s just a Birmingham thing that we have this narrative?

R: It’s the whole south, it’s (inaudible)

Mod: The south?

R: Yes.

Mod: What do you all think? Is that… is that across the board?

R: And I would say it’s not just in the south, like I’ve visited lots of other places and it’s kinda like… it’s like the norm, you know, trans women are sex workers, they’re HIV-positive, you know, they live from hotel to hotel, they’re not really stable on… you know, they don’t really have stable, you know, living arrangements.

Mod: Oh, okay.

R: You know, they… like she said most people… most trans women don’t have degrees, very few have degrees, most don’t finish… in the older generation most don’t finish… I mean high school. But, you know, I guess this is how they get caught up in the whole, um, logistics of being sex workers.

Mod: I see, okay.

R: And then, of course, along comes with sex work, sex work is a high risk… you know, that’s high risk and, you know, when you’re in sex work and you’re doing and you’re… you know, that’s your only source of income and, you know, somebody says, hey, I have $500 for you to do it bareback, you know, most people are going to go bareback and you don’t think about the long-term consequences later, so.

Mod: Yeah, yeah. Well, we know across the board we know that’s an issue when people are exchanging sex for money. I mean it’s just…

R: Yeah.

Mod: Yeah, there’s so little choice, it’s really hard. [name redacted], do you want to jump in here? You have thoughts on this?

R: Um, I can say I haven’t really… so I’m… I live in Auburn, I’m stationed in Auburn, I haven’t really experienced a lot of negative connotations. You know, I’ve gotten funny looks, I’ve been called slurs, you know, walking down the street, but I’ve never had anyone specifically point out to me and say, oh, you’re HIV-positive or anything like that. But that’s just been my experience.

Mod: Okay.

R: But, yeah, on the trans sex workers it is a very important topic because I know, I actually know a few people who have… maybe not prostitution but like would you consider like (inaudible), you know, like a porn star to be a sex worker?

R: That’s sex work to me, in my opinion.

R: Yeah… yeah.

R: (inaudible) actually having… you know, you’re not actually meeting the people in person, so I guess that’s a safer form of sex work.

R: Oh, yeah, most definitely. But, yeah.

Mod: Yeah, so that’s… that’s interesting to know. So what I’m hearing is that it sounds like the big kind of distinction that we need to think about is not necessarily trans, but it’s involvement in sex work, but I mean that’s true for everyone, but we’re… what I’m hearing you say is that a lot of people layer those together and just think, okay, trans women tend to engage in sex work therefore tend to be at risk for HIV. But you’re saying you gotta split those out because it’s the sex work that’s risky regardless of who you are.

R: Right, right, right.

Mod: Yeah, okay.

R: And then in some situations I give trans women are vulnerable to their intimate partners, just like, you know, if a partner (inaudible)… they’re vulnerable to… you know, most trans women are used to getting not loved or not… you know, just always shunned from, you know, like somebody’s not showing them, you know, a genuine love so when they finally meet someone who they… that it appears, you know, has a genuine concern or liking or loving for them, you know, they trust them and, you know, they engage in unprotected sex with these men who have sex with hundreds of trans women, they search the internet for sex working trans women, uh, they have sex with gay men, men having sex with men, they are having sex with, you know, heterosexual, you know cisgender females, so they are having all this sex and then they’re coming home to this vulnerable trans woman who’s got her feelings caught up with this person, and they’re having unprotected sex.

Mod: Yeah. But, again, what I’m hearing you say is that’s… that’s not… one of the things we’ve heard lots of people say in these interviews is there’s nothing inherent, there’s nothing specific about being trans that’s risky, but it’s all of these other situations. It’s getting involved in sex… getting involved in relationships that aren’t respectful and loving.

R: Right.

Mod: So it’s all of these other things, but we do have to be concerned about those things ‘cause we know that, you know partner violence… that, you know, violence in general is more common among trans women. So we can’t just ignore it, but it’s just this idea that it’s not… it’s not just automatic trans means HIV.

R: Right, right. And I think a lot of trans women get caught up in the sex work due to, you know, like some trans women don’t have a name change and then, you know, you’re presenting one way and then you’re going here and your name is something else. It creates a whole riff of, you know, sticky situations before you even get started with the job and then when you get started, you know, you have to encounter all these other people and they just wanna know… you know, people are gonna pass judgment on you there, so I think some trans women just feel more comfortable, hey, you know, I’ll just do the sex work, I don’t have to meet too many people… you know, I don’t have to deal with all the extra stuff, so.

Mod: Yeah… yeah. So, uh, [name redacted], you just joined us. Do you want to introduce yourself and say hello?

R: Yes, I’m sorry for my tardiness. My phone died as soon as I got back from doing what I was doing. But, hello everyone, how’s everybody doing tonight? My name is [name redacted]; I’m a resident of Birmingham, Alabama.

Mod: Good, okay. Good to meet you, [name redacted]. So just to catch you up, we’ve been talking about HIV and trans women and sort of talking about are trans women at risk for HIV, what do people in the community think, is this a big issue that trans women think about a lot? Do you have anything you want to chip in on that?

R: Yes, okay, so let me get this right… so you’re asking me… okay, repeat that for me one more time so I can make sure I have a better understanding.

Mod: We’re just talking generally about HIV and whether trans women think of themselves as being at risk for HIV and whether other people think of trans women as being at risk for HIV.

R: Well, my answer to that, yes, and the reason being is because if I’m not mistaken I got into what (name-couldn’t catch it) was saying earlier about trans women and sex workers like. I think that’s a main thing that goes through a working trans woman’s mind, especially when they’re doing that type of work. Now as for others thinking about I really don’t think that they’re thinking about it as much as they should because for some people, like that’s the only option that we have.

Mod: Uh-hum, uh-hum. Yeah, okay, so that’s going along with things that others had said, as well. Um… so sort of a related question is thinking about how… and I think [name redacted], you touched on this at the beginning, sort of thinking about how HIV and how we think of risk for HIV has changed over time. Do you feel like you hear more about HIV now or less about HIV? How has that changed, you know, since we first started talking about HIV back in the 80s?

R: Um, I guess I could start off… I guess you hear more about it. Like I said, more people are confident and, you know, willing to share their status with others. You know, it’s not so much of a clutch secret like, hey, I can’t let anybody find this out even though some people still live in that bubble, but, you know, it think a lot of people are more open, you know, living out loud, I should say, with their status.

Mod: Yeah. I like that. What do others think?

R: I don’t think it’s the death sentence that it used to be, um, back when it first initially came out. I think that as far as research and medications go there are a lot more resources and financial resources to assist with people who are living with HIV and also to prevent people from contracting HIV. I will say that.

Mod: Yeah, and that leads into one of the things that we’re going to talk about in a minute, thinking about PrEP and, you know, how people think about PrEP and whether that’s the right choice for them. But I’m curious… others, what do you think about just sort of how we understand or how we think about HIV? How has that changed over time?

R: For me, it’s more vocal. Like I get… I wanna say we’re getting more people’s attention from when we… from way back then… like people are really now starting to pay attention that this is real and it is also not a death sentence, like you can still live a… you know, a great life, it just depends up to you. And now we have more people, you know, like actually believing that and living it in that word.

Mod: Yeah, yeah, that’s true, now that we have medications where people can have near normal lifespans. That’s really incredible.

R: Right.

Mod: Back to the 80s, that was not the case, yeah. Any other comments about that? [name redacted], do you have anything you want to chip in?

R: I would just say that I haven’t been exposed to as much… I went to a private high school so I wasn’t exposed to as much sex ed as I needed to be, so I really don’t have a lot of experience with this. I’ve only ever actually talked about HIV with one person who was HIV-positive and let me know about his experience, so I’m not really familiar with a lot of the lingo.

Mod: Well, and that’s a pretty common experience, definitely in Alabama. You know, I love that you said I didn’t have a lot of sex ed; most people in Alabama didn’t have a lot of sex ed, and certainly not sex ed that really talked, you know, honestly and openly about HIV. So that’s… it’s not surprising to hear you say that. I think that’s a really common experience. So sort of using that as sort of a jumping off point for talking about PrEP, you know, Pre-Exposure Prophylaxis that people are using these days. One of the things we’re trying to figure out in this study is just what trans women think about PrEP, and whether, you know, women in your community are thinking that getting on PrEP is a good idea or not a good idea. So that’s kind of what I want to shift to focus on now. What are you hearing about… about people and how they make decisions about whether PrEP is the right thing for them?

R: Um, I’ll start off with this question. When it comes to PrEP I don’t think trans women were thought about when the drug was created, that’s my personal opinion. The reason I say that is because it has such a negative effect on us as far as it relates to us and our HRT. Now, what I will say, it is definitely pushed a lot here in Alabama, as well as south, but I think as far as agencies pushing and trying to get patients on PrEP I don’t think they’re keeping in mind of the other effects that it has on trans women, more so just for them to get numbers, to say, oh, we have X amount of patients now in our PrEP clinic. So I don’t think… my person… I personally decided not to take PrEP because of the negative effects that it would have on my HRT, so I did not take it. However, I do know some trans women who are taking PrEP and that’s their choice, but I just think when it comes to PrEP I think a gay man, a gay cis man was thought about it when it came time for the drug to be designed and created.

Mod: Okay. So I’m curious, you know, it sounds like you’ve thought really carefully about this and done some research. Where did you find information that helped you to make the best decision for you about, you know, you’re talking about the way that PrEP and HRT might interact. How did you… how did you learn that?

R: I had a friend who was working at a PrEP clinic here in Birmingham and he brought that up one day in conversation and just recommended that I be on it. And I’m the type of person who I’m just not gonna decide to take pills and not know why I’m taking pills; that’s me personally. So I had a conversation with my doctor who does oversee my HRT treatment and she told me that I could take it but she said did they go over the effects that it would have on your HRT, and I said, no, nobody ever brought that up. All I got was that they were going to test my liver, this, that and the third, and, boom, I would have my medicine. They didn’t care about whether I may possibly be allergic to it or not, so it’s just why… that’s why I have the experience that I have with PrEP, is I just feel like at this point it’s numbers and not more so about the individual who is possibly gonna be taking this drug.

Mod: Okay. I mean I have to say I love that you talked to your doctor about that and did you feel like your doctor was knowledgeable and able to answer those questions?

R: My doctor, she also prescribes those type drugs to some of her patients. She doesn’t only deal with trans women; she deal with other patients, as well. So, um, that’s why I consulted with her. She told me I can take it but she said that… like I said, it would have a negative on my HRT, so I decided not to take it.

Mod: Well, I have to say I’m really excited to know that you talked to a doctor who you felt was knowledgeable because we’ve heard a lot of people say, you know, I tried asking a doctor and they’re like, yeah, sure, you should take it, it’s a good idea and so it’s… that’s… that makes me feel good that you had a good experience talking to your doctor and that you felt like it was informative. What are other… what are other thoughts on PrEP? What are you hearing about PrEP out in the community?

R: Um, I know… I know lots of people who are on PrEP and I know some trans women who are on PrEP, I know some, you know, just people in the community on PrEP. Actually about two years ago my doctor was, you know, recommending it for me. I passed because I’m, you know, at this age I’m not sexually active anymore, you know, so I don’t… it think to me, if I had had the PrEP option maybe ten years ago it probably would have been to me like, okay, I can go do what I wanna do. But, you know, I’m not sexually active now so I don’t, you know… PrEP wouldn’t be a, you know, a thing that would be useful to me because I don’t engage in those activities, but I… I do hear a lot of people pushin’ it, so, yeah, I hear about it all the time.

Mod: And when you say you hear about it all the time, what are… like do you mean you hear about it out and about in the community?

R: Um, I hear some… you know, just like in normal, you know, house settings or focus groups or whatever, I hear some good things about it, I hear some bad things about it, some people that I know in my inner circle do take it. Some have good experiences, some have bad experience.

Mod: Yeah.

R: And my doctor, he mentioned it to me like that one time and then for my six month with my HRT appointment, you know, like it was… he mentioned it those first two times and after he saw that I wasn’t really, you know, responsive to it, receptive to it, he hadn’t even mentioned it to me again, so.

Mod: Okay. Other experiences in hearing about PrEP or thinking about PrEP?

R: To be honest, I didn’t even know PrEP was a thing until I learned about this study.

Mod: Wow. Okay.

R: Oh, wow.

Mod: Well, that’s great! So we’re doing some education here. (laughs) I love it, yeah. You mentioned earlier that you were down in Auburn. Have you always been down in Auburn or did you go down there from Birmingham?

R: So I fluctuated between Birmingham and Montgomery when I was growing up.

Mod: Oh, that’s right, because you said (inaudible). Okay, well, that’s important to know, too, that, you know, not everyone is hearing about PrEP equally. Yeah, that’s good to know.

R: Yeah. But it’s also interesting because I’ve had multiple partners who were trans women, too, but they’ve never said anything about it at all, so that’s also interesting. You know, again, that’s just my experience, you know, just, you know, we all have different experiences but it’s just my experience, yeah.

Mod: Yeah. Well, that’s… I mean that’s why we’re doing the study, is to hear everyone’s experiences so that’s really valuable to hear. Okay. Anyone else? [name redacted], did you want to chime in?

R: Uh, yes, for the moment I am actually on PrEP and it kind of shocked me when… I think me and Desiree had discussed what she had explained about PrEP not too long ago and it kind of shocked me that I found out about that. So, I don’t know… I guess I might end up considering about, you know, dropping PrEP, you know, just to play it safe with my HRT.

Mod: Yeah. So that’s a big decision. What are you, like what will you think about and how are you… how will you figure out what the best thing to do for you is to be?

R: Well, me personally, I feel like I already know what the best option for me is going to be because the goal for me is to become… become my authentic self. And I don’t need anything that is going to, you know, distract me or, you know, not let me get to my authentic self. So I don’t need any blocks in the road so I’m pretty sure what I’m gonna do in the long run.

Mod: Okay. That’s really… that’s really critical information. And honestly that’s… sorry.

R: I’m sorry. One other thing for me, it was kind of a no for me because like, well, I’m forty, so I made it from eighteen all the way to forty negative and, you know, I’ve done risky behaviors, I’ve participated in sex work, but I’ve always kind of had that.. my own personal safety net I, you know, kind of live by, that’s why I was kinda like, you know, I don’t really need it, I didn’t really see myself takin’ it.

Mod: Okay, but so it sounds like for you it’s sort of also an age thing. You feel like that…

R: Yeah, I feel like I made it all the way here…

Mod: Those behaviors (inaudible-talking over)…

R: Yeah.

Mod: Yeah, okay. That’s… that’s really interesting. Um, so, now I’m curious to learn more, and we’ve talked about this a little bit, but, you know, what… what are some different resources… you know, several of you talked about hearing directly from doctors and that is so relieving to hear that you feel like your doctors have been helpful in that. What are some other sources that you hear about PrEP from?

R: Um, like I said, I have a friend, um, two friends, one that no longer works with the agency, but I do have a very close friend of mine who works with the PrEP clinic, actually works with [name redacted], but, um, when I had the conversation with him… ‘cause he’s my best friend, but I call him my brother but just because how long we’ve known each other for. I had the conversation with him. He told me, he was like I personally wouldn’t recommend the drug for you because you’re not a… he used the term ‘health sexual’ (laughs), so he was like you’re not a healthy sexual so I don’t see the need for you to take it. And I’m not… I’m not… I have sex but I only have sex when I’m in a relationship, let me clarify that. So if I… if I were to become… engage into a relationship with somebody and I probably feel like I need to take those risks then I would, but as of now I don’t see the need to.

Mod: Alright. So what I’m hearing people sort of say is it also has to do with where you’re at, sort of your relationship status and what choices you’re making right now about any risk behaviors that you might engage in, and so it’s rather than, you know, thinking just about PrEP, it’s also thinking about the context that you’re currently in that’s, you know, that plays into it as well. That’s really interesting. What about… um, one of the things that we’ve heard lot of people talk about is ads that they see about PrEP. Have you seen any ads talking about PrEP?

R: You see ads, but like I said it’s just gay men in those ads. You rarely… I know, for me, I’ve never seen a trans woman in a PrEP ad, so, um, that’s why I said it was… trans women weren’t thought of when that drug was created, because it’s like you never see trans women when it comes to advertisement for PrEP. I know I haven’t seen any. I don’t know if [names redacted]may have seen…

R: Yeah.

R: Go ahead.

R: I’ve seen.. I can’t remember… you know, when (inaudible) comes on, like kind of late night television when they’re advertising all the different stuff, but I did see a trans woman on there and then I guess someone was, you know, up in drag and it says not recommended for people born female at birth, or something. And that has caught my attention. But I did see… that was the first… the only one that I’ve seen with the trans woman and the other person dressed up in drag on there. But all the posters that I’ve seen about PrEP is, you know, you know, men.

R: Yeah, and I can honestly say that I haven’t seen one that’s (inaudible) trans women here… well, in Alabama. Now in Atlanta, of course, I’ve seen it on billboards, buses, you know, it’s just I haven’t seen it in Birmingham.

Mod: Why do you think that’s different?

R: I guess more of the population, like, yeah, probably that.

R: Yeah… yeah, Georgia’s a bit more of… not quite as conservative as Alabama.

R: Uh-hum.

Mod: I think that’s fair to say (laughs). Yeah, everyone’s nodding, yeah.

R: I think there’s a lot of trans women in Alabama that, you know, they do their own thing, they live their own life. You know, they’re not like on the Richter scale. ‘Cause like I live in Birmingham and I never met (inaudible), but I don’t frequent clubs and, you know, all these other spaces and, you know, (inaudible) and I’m thinkin’ the same thing, so, yeah, I think it’s a lot of trans women that do live in Alabama but they just, you know, just livin’ themselves, mind their own business and stay to themselves and, you know, stay off the radar of things that are goin’ on, I guess.

R: I’m gonna have to agree with her. I know for me, I mind my business…

[Laughter]

R: … um, out there… if I hadn’t been part of this study I probably would have never seen or met like other women. I know [name redacted] personally, so [name redacted]’s the only trans woman besides another trans woman that we mutually know that lives here. So but it’s also gonna be a culture shock (inaudible) I encounter, just found out I’m gonna have to move to Nashville soon, so it’s gonna be a culture shock to see… yes, Karina, I didn’t tell you yet, don’t get mad, don’t show that on the Zoom call.

R: I’m not. I’m not.

[Several talking over each other/laughing]

R: It’s gonna be a culture shock to see how many trans women I encounter in Nashville.

R: There’s quite a few.

Mod: Yeah, okay.

R: To me in Alabama it seems like it’s a second level of being in the closet, if that makes sense.

R: It is.

Mod: Tell me more.

R: So to me like I… you know, I grew up, I knew a lot of people who were gay, I knew a lot of people who were lesbian, a lot of people who were bisexual, but unless they had specifically told me I didn’t know they were trans. I didn’t meet my first trans person until I was a sophomore in college honestly. So it… so, now I had probably ran across someone who was trans, I just didn’t know it.

R: Okay, yeah, that’s what I was about to interject and say.

Mod: Yeah, may not always know.

R: Right, right, right.

Mod: Um, so this has been… this has been really good. Is there anything else? I said at the beginning I’m counting on you to tell me what are the right questions to be asking.

R: Is that all the questions on that list already?

Mod: Yeah, I told you it would go fast. You’re like, oh, we’re gonna be here four hours with all her questions (laughing).

R: Well, how many questions was it?

Mod: What, no, I just like flashed my list. You can see I’ve been writing notes and stuff, whatnot, yeah.

R: Okay, okay.

Mod: Yeah, no, I’m good, I make it feel like a conversation.

R: I just… I just… I think the only message y’all would want to send out to the world about trans women is that we… we all don’t live by the preconceived notion, pre-written narrative that society has written for us. We’re all different in our own individual ways. Just ‘cause I have a college degree doesn’t make me better than the next trans woman, I just chose another route. But I have met some successful trans women who have started their own businesses, they’re owning non-profits. So I think that would be the message that I would like to send out to the world, that we’re all not living by that pre-conceived notion or what society thinks that we are.

Mod: Yeah, yeah, that’s important. Any other…

R: Like I said at the beginning of the call, like when you meet, I guess, you know, like, of course we all approach these (inaudible) searching for conversation, you know, even if we don’t do anything else, just good conversation, rumination. It’s like you meet people and, you know, they’re like do you do sex work or, are you… do you know your status, or do you do drugs. It’s kinda like all these things kinda like come along with… okay, just because I’m trans doesn’t mean I do hard drugs, doesn’t mean I do sex work, doesn’t mean I’m… you know, just kinda like a whole laid out book, you know, where the conversation is going, okay, hey, I wanna take you out, but when you say, hey, I’m trans it’s kinda, hey, do you do hard drugs, do you do this, you know, do you do sex work… it’s kind of… goes in a different direction.

Mod: And do you feel like that’s… oh, I’m sorry.

R: No, go ahead. Go ahead.

Mod: I was just going to ask do you think that… like do you feel that mainly from cis from people and from hetero people or do you feel… do you get that in the LGBTQ community, too?

R: Uh, a lot of it goes on in the LGBTQ community. I think a lot of it goes on there, as well.

Mod: Okay.

R: And then I even, you know, just doing my own little thing with like guys and me, I’ve even told ‘em, yeah, I’m positive but that still doesn’t change, like, okay, they still wanna have sex, they still wanna have unprotected sex. So like, you know, it’s kinda like, you know, like wow, so.

Mod: Yep. Okay.

R: I guess that was a little study of my own.

Mod: [name redacted], were you going to say something?

R: Yeah, so you were talking about the, um, a lot of the negative connotations that are associated with trans people when it comes to drugs and everything, and, yeah, I’ve definitely… now this is just the circle of people I’ve experienced… you know, I really haven’t experienced the older generation of LGBTQ peoples so I’ve really only dealt with the college age, so I, um... but, yeah, cis people... a lot of cis people just don’t get it, you know. They try. You know, I’ve had a lot of people try but they just do not get the fact that just because you’re trans your… you know, you’re not out there partying, you’re not, you know, a sex worker, you know, you don’t do hard drugs just because you’re trans, you know.

Mod: Yeah, yeah.

R: One of the ways to change that is by educating people. I know I’ve had to do some diversity and inclusion training in my office because we hire at a high volume in my job so we’re talking like 800 people a week that I’m looking at in my office on a day to day basis. So we had a couple of trans women who came, trans women and trans men who came into my office and I had some colleagues who just did some outrageous things and I’m like this is just not okay. So I didn’t want to, per se, get the pitchforks and stuff out at my job. I went about it another route and come to find out a lot of… all of them never had any issues with trans people or the LGBTQ community. They were ignorant, and I’m using ignorant because they were uneducated about how to deal with people who identify with that identity. That was the shocker. And then what was more shocking is none of them even knew I was trans, not that that was something that I had shared with them, but when I did share that it was like, what… like wow. (laughs) So I think education is another way to change (inaudible) So I’ve been tryin’ to do that even at my school. I went to a historical black college and a lot of our administrators were old school, like born in like the 70s and…

Mod: (inaudible) (laughing)

[LAUGHING]

R: Okay, 60s. Great, I like that better. So, um, yeah, a lot of them were older and set in their ways and a lot of them were like ministers and things of that nature, so a lot of them were discriminated against within our community and so I had to go in and do trainings there, especially with our housing department. Our housing department still was housing trans women in male dormitories and that was just not okay. So I think, like I said, education would be one way to change the narrative and the way that our community is being treated.

R: They also have to have the mindset to be willing to be educated, most definitely, to receive the education.

R: Yes, yes.

Mod: Yeah, and that’s… that’s the hard part because we can’t control how other people are willing to engage, but, yeah.

R: Just like public school, do they have like a section on LGBTQ health or is it just like… I don’t know.

Mod: Yeah, probably not in Alabama. Some states have more comprehensive sex education that talk about, you know, a much broader range. I mean in Alabama I don’t believe they’re really supposed to talk about sex outside of heterosexual marriage. So that sort of… that takes a lot of things off the table, you know.

R: We didn’t even have sex ed when I came from high school. I’m from (inaudible), Alabama, and that is like the most like rural part of the state, and we didn’t talk about… the only thing that we talked about that was any relation to sex was in the fifth grade when they talked about puberty and it was like girls, this is what happens, blah, blah, blah… men, this is what happens, blah, blah, blah. They gave the women a stick of deodorant and a maxi-pad or tampon, sent them on their way, and gave the boys a stick of deodorant and sent them on their way, and that was it. It was just that was it, you didn’t hear about sex no more.

Mod: Yeah.

R: Yeah, ‘cause I went to school in Selma and I didn’t… I was trying to think back and I don’t… my sex ed class, it was all about, okay, this is the female anatomy and this is the male anatomy. It wasn’t anything sexual talked about, it was just, you know, if you’re male you have this and if you’re female you have this, and that was, you know, what you do with the two was never talked about.

Mod: No… yeah. Yeah, I went to school in Virginia in the 80s, if you can believe that.

R: So you were born in the 70s (laughs)

Mod: I was in middle school in the 80s, yeah, and that was it. But, you know, that’s really common because that’s been a huge political fight for years, is abstinence only versus, you know, comprehensive sex education.

R: And the thing that we’re paying for is that in Montgomery we have one of the highest rates of STD… we have one of the highest STD rates in the… in America, at least… I don’t know about the world, but definitely in America. And…

R: Thank you, [name redacted]! (laughs)

Mod: Yeah, it’s stunning, but, you know, and it… and it maps very well where STD rates are high is where comprehensive…

(inaudible-talking over)

R: … we have a lot of colleges and universities there, Alabama State included, AUM, Faulkner, Huntingdon… like all of those schools make up a large percentage of those numbers that Michele just disclosed.

Mod: Yes. Even at the high school level, you know, one in four high school students gets diagnosed with an STD. I mean it’s just, yeah, the numbers are…

R: That was a culture shock to me when you would hear students contracting STIs in high school. I was like oh my god.

R: Why was it such a shocker to you, if you don’t mind me asking?

R: I couldn’t… my home town is very conservative, so when you hear STIs it’s like you might as well sew like a scarlet letter on your sweater because you’re just labeled a slut or a whore, that’s just what you’re gonna be labeled as. But like it was a culture shock to hear like kids my age contracting STIs; it was just like oh my god, where’s your mother, let me call your mother. (laughs)

R: I can remember in my life, like eleventh or twelfth grade, sex ed class it was like five pregnant girls in the class, so there was some sex goin’ on somewhere. Maybe, you know, they just weren’t talkin’ about it but it was… I can remember like very vividly there was like four or five pregnant girls in the sex ed class and they weren’t talkin’ about any sex, just this is male and this is female. Well, clearly they had combined because we had, you know, these people who were pregnant in the class.

Mod: Yeah, someone had figured it out, hmm, yeah.

R: And it really is a problem because increasingly we’re sexualized at a younger and younger age, you know, and you get exposed to it at a younger and younger age every time and even if you have it in the twelfth grade most people have had… well, I don’t know about most people, but a lot of people have had sex by then.

Mod: Yep… yep.

R: (inaudible)

Mod: Yeah, yeah. No, it needs to be… well, obviously, I have strong feelings about this, this is something that I work a lot on, but yeah, early comprehensive sex education… it works. And it’s not just important… you know, I mean we’re talking about STIs and pregnancy and that sort of thing; it’s important for all sorts of things. It’s important for knowing how to negotiate boundaries and relationships. We talked earlier about people being in relationships where they didn’t feel like they could, you know, say no to things, and that’s, you know, that’s part of what good comprehensive sex education provides people with, is ways of talking about, you know, what feels safe and what’s a reasonable thing to do and how you talk with partners about that, so yeah. I could go on and on, but we are actually… oh my goodness, we’re coming up on the hour. Are there other things that people need us to know? What else do you want us to hear? Anything?

R: Nothing that jumps right out at me at the moment.

Mod: Yeah, I feel like we’ve had a really good comprehensive discussion.

R: I was curious… I was curious… y’all don’t have to answer me if y’all aren’t comfortable, but how is being an African American trans woman? Because, you know, I’m white but I know y’all have certain difficulties because you get it double… you know, you get racial profile and then you get profiled because of that, so how does that compare?

R: That would be like walking a fresh pair of (inaudible), your feet are gonna hurt, but we make it do (inaudible).

R: Um, and I’ve had some problems. I haven’t had a lot of problems. I did when I was younger and I was starting out when it wasn’t, I wasn’t so easy in passing, I have a large family, so that kind of helped me, too.

R: Okay, I just wanted to… I know I’ve read a lot of statistics on… I’ve read some statistics on it and like it… like it’s… aren’t African American trans women the most likely homicide victims in…

R: Right.

R: Yeah.

R: The average age is like thirty-five, I think… thirty-four, thirty-five.

R: Yeah, that’s really unfortunate. I’m sorry… I’m sorry if I tread…

R: No, no, no… and one other thing I, you know, like just evaluated my friends, my (inaudible) friends down through the years, like she said support system. When you have girls who have, you know, affirming support system their friendship level… you know, their everyday (inaudible), you know, maintaining in society is gonna be different versus someone who grew up or their families shunned them because they have a whole different, I guess, like a survival skill, you know, their survival skill has kicked in. And, you know, it’s hard to be friends with people who have those survival skills, I guess. So I guess like she said, an affirming support system.

R: Okay, yeah, I get that.

Mod: Alright, well I want to be respectful of time. Any other comments before we breaks? Alright, well, thank you.

R: Nice to meet everybody.

Mod: Yeah, so nice to meet everyone. I really appreciate you taking the time, especially this week. This may be a challenging week for a lot of different reasons. Hopefully it’s a good week, but in any case… yeah, Dr. Van Gerwen, do you want to hop in and wrap up?

Mod: Yes, I just have one… Hi… I just have one housekeeping comment. As far as reimbursement goes I think I’ve talked to all of you individually. We have VISA gift cards for you all for participating and I’ll be in touch with you individually about how we can exchange them because we’ll either need to meet in person or I’ll need to figure out a way to mail them to you if you don’t live in town. So I’ll be in touch with each of you individually tomorrow. But, um, I’ll echo [name redacted] and say this was a great focus group and I’m glad everyone was able to attend and we appreciate everybody’s insights and participation. And so, I just want to make sure everybody knows the first part of the work is understanding, you know, the issues that face this community but the most important part of the work is using this information to help take better care of trans women and all patients in the sexual health field. So that’s why we do this work, so we can take better care of our patients. So we can use your perspectives and your insights and your lived experiences to inform that care, so we hear you and we want to use this information to help make your lives better. We appreciate it. Alright, thanks, everyone.

Mod: Thank you, ladies.

[Everyone saying goodbye]

END OF RECORDING
