# Supplementary material for: “It’s behaviors, not identity”: Attitudes and beliefs related to HIV risk and pre-exposure prophylaxis among transgender women in the Southeastern United States
Source: PLoS One. 2022 Jan 27;17(1):e0262205. doi: 10.1371/journal.pone.0262205 (PMC8794203; doi:10.1371/journal.pone.0262205)
Supplement: S4 File — (DOCX) [file pone.0262205.s004.docx]

Van Gerwen

HIV Study

File Name: File 4

Length of File: 54:05

… this evening. My video won’t be on and I’ll be muted because I have a chaotic puppy and baby in the background, so I’m gonna mute myself but I’m listening and I’m here.

Mod: Great. Thank you, Dr. Ventro. And so, as Libby said, I’m Dr. Austin. I go by Ela, it’s my chosen. It happens to be my initials, so it works out really well. And first, before we get started I’d love for each of you to introduce yourselves so I can hear how you say your names. Can everyone do that?

R: Yes. Hello?

Mod: Are you able to turn on your cameras? It’s okay if you…

R: It’s on.

Mod: Okay, so who all do we have with us tonight?

R: (inaudible)

R: I’m [name redacted]. Nice to meet everyone. How are you?

Mod: Hi [name redacted]. Good to meet you. I’m glad you’re here.

R: I’m [name redacted].

Mod: [name redacted], good to meet you.

R: Nice to meet you, too.

Mod: Pretty name.

R: Thank you.

Mod: And [name redacted].

R: Yes.

Mod: Good to meet you, as well. Okay, so, Dr. Van Gerwen has talked a little bit about what we’re doing in the study and we’re actually in the process of kind of wrapping up this study, so all of you tonight are going to play a really important role for us. We’ll start off the focus group by just talking through some of these issues and hearing your ideas, but then I want us to save a little bit of time at the end of our focus group and what I’m going to ask all of you to do is to help us reflect on some of the things that we’ve heard from all of the other women that we’ve spoken with in this particular study. So Dr. Van Gerwen has gone through and has read through over and over again all of the transcripts of the focus groups that we’ve conducted previously and has started to think about what are the common things that we’re hearing as we’re talking to transgender women out there, views on HIV and sexual health and these sorts of things. And she’s kind of made a list of the top things that we’ve heard but it will be really helpful for to sort of turn that back around and say this is what we think we heard, does this sound right… are we understanding this correctly. So we’ll save some time at the end for that because that’s (inaudible-loud noises) figuring how we can better serve transgender women in Birmingham. (loud noises) have all of your voices and really reflect and make sure that we’re understanding things correctly. So that’s going to be a really important part of what we talk about tonight. Before we get started with that I kind of just want to talk through general issues, sort of how transgender women in Birmingham… and here what’s nice is that you can talk about your own experiences, your own ideas, your own thoughts on these things, but also we’re really hoping to hear from all trans women in Birmingham, so feel free to say we know I’ve had this experience, but a friend of mine has had this experience, so, you know, we’re really looking to hear as many voices as possible. So don’t feel like you only have to say, oh, well, I don’t know anything about that, I’ve never heard about that. We’re really looking for your voices, friends’ voices, trying to get everyone kind of in the room. So the first thing I want to talk about is just sort of how trans women in the Birmingham think about HIV. Is this something that people talk about a lot? Is it like not ever mentioned? What’s the current discussion around HIV among people that you know in your community? Who wants to start?

R: I guess I will.

Mod: Okay, great.

R: So in the groups I hang out with it’s not usually talked about unless the subject of sex comes into frame. In that case, it’s usually a supportive thing, like someone says, oh, I’m starting a relationship and then you kind of just ask them have you guys gotten tested yet, you guys wearing protection or taking precaution so that if something happens you’re able to seek medical attention, and kind of supporting each other and keeping tabs just to make sure people are okay and keeping that in their frame of mind when pursuing relationships at that… in that moment and to that extent.

Mod: That’s helpful. It’s good to have friends who are looking out for each other like that. I like to hear that.

R: Absolutely.

Mod: What do others think? What’s been the experience of hearing about or talking about HIV among people you know in your community?

R: Well, for me, I have not ever had a discussion about HIV amongst like any of my friends, nor relatives. It’s not even like a topic. Like even if we’re talking about sex or intimacy that is never a discussion, like, oh, this person had an STD, like that never comes up. It’s like… it’s kind of like they brush it off as if it don’t exist.

Mod: Hmm… do you feel like people intentionally don’t talk about that because it’s not something you’re supposed to talk about or it’s just not something that people are even thinking about? Can you tell which… which it is?

R: Um, definitely the first way. I feel like it’s just people don’t wanna talk about it. They try to avoid it. But I know people… I have come in contact with people who have HIV and I really didn’t know how to handle it, but I’ve grown from the two times that this has occurred. But I would like kind of, um… distance myself from them, like I may not respond to the message or, you know, if this was a person’s whose house I was goin’ over and we were like watchin’ movies, not even bein’ sexually active and it came to that point of the person wanting to do that and then they told me that I was like a deer in the headlights, like I didn’t know what to do. So I know that it’s real ‘cause I’ve had two different occurrences with that, but it’s like I said it’s never a topic amongst others.

Mod: Yeah, yeah. Was… do you feel… was that a recent occurrence or were those long time ago?

R: Uh, that one happened in 2019 and one happened in 2018.

Mod: Okay, so pretty recent, yeah. Yeah, I think that’s a really common sort of experience. Even though, you know, we may hear about HIV a lot we sometimes, you know, like you said, deer in the headlights, like oh my gosh, what am I supposed to do here, what’s the right thing, what are the things… you know, where’s [name redacted] and her friends who were talking about the right things to do. You know, I think that’s a pretty common experience. Other thoughts about HIV that you hear people talking about?

R: (inaudible-breaking up) Hello?

Mod: We can hear you, [name redacted]. Go ahead.

R: (inaudible-several people talking/background noise) Hello?

Mod: [name redacted], can you hear us here on the…?

R: Yes.

Mod: Okay. Did you want to say something about thoughts on HIV?

R: Well, yeah, I know my friends have it and always talked to them about it, to stay positive and take their own PrEP.

Mod: Oh, no. Are you back in?

R: Yeah, I’m back.

Mod: Okay. Yeah, you’re cutting in and out a little bit but I want to make sure we’re capturing this. So you say that you do have… you have some friends and you talk with them about HIV?

R: Yes.

Mod: Okay. So I’m also curious, and any of you can kind of speak to this… and I can’t remember who was sort of touching on this earlier… can how we talk about HIV or how we think about HIV has changed over time? I mean I don’t know how old any of you are. I’m certainly old enough to remember when HIV first started and first was on the scene and certainly, you know, all of us are old enough to remember when HIV started to really become a major issue in the southeast because, you know, that wasn’t always the case and it’s only been, you know, much more recently that here in Birmingham and Atlanta, New Orleans and stuff that we actually think of HIV being a big issue. Do you feel like how people have talked about HIV and thought about their risk of HIV has changed over time or what’s been your experience with that? Like sort of thinking back to now, how has that changed?

R: For me most of the people I know are much more aware of sexual diseases in general. Before it was mainly in high school and that you talked about the reproductive system, you talked about all of that, but the sexual diseases were kind of like an afterthought, something that would be mentioned but not usually in-depth, or only be for a single day. Now this time period seems like it is still a little bit of an afterthought and a taboo to talk about it in general conversation, but once the subject of sex comes up it seems to me to be more easier or, um, more now recognized as an actual thing that happens and should be talked about rather than something to hide, something to be ashamed of, or something to degrade others for.

Mod: Hmm, so it sounds like progress from what you’re describing, so that’s good. Right? Good news. What are other thoughts about sort of how we think about HIV has changed over time?

R: Um… I really don’t know, to be honest, because I’ve never… I wasn’t taught… like growing up I wasn’t taught about sex. There never was like a topic in my family, so I really didn’t start learning about these type of things until I got older and began to, um, you know, become more sexually active and different things like that, so you learn as you go on. But as far as growth I don’t know because I really haven’t known about it until not too long ago, like the last four or five years.

Mod: Yeah. So I’m curious, you know, because this is something we keep hearing… no one hears about these things in school, no one talks about these things in their family… what… where have you started to learn more and to hear more about HIV or other sexually transmitted diseases? Like what have been good resources for learning about that kind of stuff?

R: Um, going to my doctors. I go to a lot of different doctors, so they keep me informed about a lot of things when they have to draw blood and different things, I know they always ask those questions. I had to have a procedure done where they actually had to make sure I was HIV-negative and everything in order to do it, basically to, you know, preserve some things for my future, so I have children and different things like that. So my doctors just kind of keep me up on everything.

Mod: Okay, so that’s… that’s a good source of information, likely to be reliable, so that’s good. And it… you know, it sounds like you have good communication with doctors. What are some other sources that people draw on since we know that, you know, high school sex education is not always as detailed as we probably need it to be, or as inclusive.

R: My high…

R: (inaudible) Oh, go ahead.

R: No, you can go.

R: I was going to say, my high school, we didn’t really learn. Our teacher was a football coach so we didn’t really kind of learn about that type of stuff, which is horrible to say, but in high school that’s just like what happened.

Mod: And I think that’s a really common experience, so you’re not alone in not having had great education on that, you know. What were you going to say, [name redacted]?

R: Um, one area that I find that led me to find more information about it was just looking up LGBT or Pride material in general. A lot of areas in that do mention, um, HIV and other sexual transmitted disease because that was one of the groups that got hit the most by it during the early years. And that way, when you look at information for transitioning medically or non, it’s usually brought up, it’s usually talked about or shown through pamphlets or informational briefs, LGBT areas and websites and media.

Mod: Okay. So you found that to be a good source, too?

R: Yeah, kinda like a media osmosis, if you will, where you kind of get a little bit of snippets from this poster, a little bit from this website, usually when you’re not even directly looking for them, but you kind of just see information.

Mod: Interesting, they’re just kind of showing up, you keep kind of taking that in. Hmm. Well, I bet that’s effective. I bet you know all sorts of stuff that you don’t even know that you know. (laughs) So that’s cool. So the next thing I want to talk about, sort of following up on a lot of the things that have been brought up so far is thinking about PrEP… you know Pre-Exposure Prophylaxis. And this is something that, you know, has been really popular in recent years. Lots of people are adopting PrEP, lots of health care providers are recommending PrEP, but one of the things that we continue to see is that the use of PrEP among trans women is still really low, and so one of the things that we were trying to figure out in this study is why is that, why has PrEP not been adopted more widely among trans women. So that’s the next kind of big topic that I’d love to get your thoughts on… you know, sort of where do you hear about PrEP, what are your thoughts on PrEP, what are barriers to PrEP, what do you see as benefits, all of that. That’s a lot of questions. (laughs)

R: No problem. For me, I’ve actually never really heard of PrEP in detail. Again, it’s been like scraps, but specific in this area I think I’ve only heard PrEP given to me once as like a general kind of descriptor of what it was and then I don’t think I’ve heard it from areas since. Like I’ve heard specifically about HIV and that kind of stuff, specific disease and ways of dealing with it like protection, visiting your doctor, that kind of stuff. But I haven’t really heard much about PrEP as its own thing.

Mod: Okay, so that’s good to know, that’s exactly the kind of information that we’re looking for. So we’re… you know, we’re asking questions about why don’t people, you know, use PrEP more, and you’re telling me because we don’t even hear about PrEP. So that’s… yeah, that’s exactly the kind of feedback that we’re hoping to get in this study, so that’s really helpful. [name redacted], do you have thoughts on that?

R: I do. So, socially I heard about PrEP, ironically, from a club, like there’s a club in Birmingham and it’s a gay club, and they actually have like different promotions and like you get in you get five dollars off admission like if you sign off for PrEP, and they have like, I guess, the lady there that takes your information. But I was just like (inaudible) ironic on the social scene, and then also, of course, talking to my physicians about PrEP as well.

Mod: Okay, so interesting, like out at a club that you…

R: Yes… ironically, yes. So they would actually have fliers… they would make their fliers and it would have the promotion for if you… you know, if you sign up for PrEP.

Mod: So how did you feel about that, ‘cause that’s… you know, that’s something that we always talk about, like, oh, maybe we could advertise things at the club and some people say, yeah, that’s a great idea and some people say I’m not trying to think about that when I’m out trying to have a good time, and so we get really different opinions. I’d love to hear your… your thoughts. Did you feel like that was a good place to advertise that?

R: I do. It really was an eye opener because I looked at it like, okay, stuff is really (inaudible). The club owners, where you’re going to turn up and socialize with a lot of people… you know, if the owners of those clubs are promoting PrEP something to, you know, to be able to take to cover yourself, that was a big eye opener for me, so I was like, wow.

Mod: Okay. So you saw it as a positive.

R: I did.

Mod: Okay. Well, that’s really interesting to hear because that’s, you know, every time we ask people we get different opinions on that. So, [name redacted], I see that you’re back. We’re talking about PrEP and sort of how people hear about PrEP, what people in the trans community think about using PrEP. Is there anything you want to share about that?

R: Yes, um, I’m on PrEP myself. I just heard it through the TV.

Mod: I’m sorry, what?

R: I said I heard PrEP… about PrEP on television.

Mod: You heard it like on an ad or a TV show or what?

R: A commercial.

Mod: Okay. And like what did you think about that commercial? Did you feel like it was speaking to you?

R: It was, and I’m taking it. So I take PrEP. I take PrEP for two reasons, you know, because (inaudible) someone and I don’t wanna try to get nothin’.

Mod: Uh-hum. Yeah, okay. So that’s interesting that you said you saw a TV ad and that that’s where you got the idea. Was it clear to you once you saw that ad, what you needed to and where you need to go to get access to PrEP?

R: Yes. Hello?

Mod: Yeah, we can hear you.

R: Uh-huh. See, watching the commercial was a wakeup call for me.

Mod: Yeah? And then you were able to figure out where in the community you could go to get access to PrEP?

R: Yes, health department.

Mod: Okay, cool… so you knew what to do once you saw that TV ad.

R: Uh-hum… yes.

Mod: Okay, well, that’s good to hear ‘cause that’s something that, you know, and we’ll talk about that in a minute, that we’ve heard sort of differing ideas on how useful ads like that are. I am curious about are there any particular barriers that you hear about that, that maybe you’ve thought about yourself and that you’ve heard other trans women talk about like why they wouldn’t want to go on PrEP? Is this something that people talk about really? What do you think?

R: So for me, once again, it’s not really a topic, but some of my trans friends that I know, um, they actually are already on PrEP, so like they love PrEP and they’re always telling me I need to get on PrEP But I feel like as far as… I don’t know, I feel like the resources for trans women and trans people in general are not there, especially in Birmingham. I feel like they don’t advertise stuff as much as they should… uh, yeah

Mod: Yeah, and that’s a really common theme you’ll see in a second when we talk about kind of what we’ve heard from all these different focus groups. That’s a really common feeling that people have, that, you know, maybe PrEP’s a great thing but we don’t really hear about it and we definitely don’t hear about it, you know, talked about from the perspective of trans women and why it might be useful or, you know, what some of the considerations are. So it… it sounds like that’s a pretty common… pretty common view. So before we move on to the second half where we kind of talk through some of the themes that we’ve heard is there anything else that you all need me to hear about just HIV, PrEP, all of these different things that we’ve talked about so far? Any big… big ideas come to you as we were talking? Okay, then… yeah, [name redacted], go ahead.

R: Not for me. Um, like I said, I just feel like we need more resources, and then it takes forever, which is still a lack of resources when it comes to getting on PrEP. You know, you have to wait like months down the line in order to even get it. So I feel like that’s horrible, too.

Mod: So just health care access in general, it sounds like…

R: Yes… yes.

Mod: … you’re pointing to as an issue, yeah. And that’s a consistent concern, even though, you know, we work hard and try every day opening up new opportunities, but it’s still… it’s hard to get care, and that’s sort of across the board, so, yeah. So what I want to do now, like I said, is kind of talk through some of the themes that we’ve heard so far. And what I’m really looking for is just for y’all to say, yeah, I can see that people would say that, even if it’s not necessarily how you view things. But just kind of, you know, making sure that we’ve understood from all of these focus groups that we’ve done, you know, that we’ve listened to. I’ve moderated and I’ve been, you know, kind of, you know, listening and trying to put together a, you know, a story of what people are telling us. But we want to make sure that we’ve told that story correctly. So what I want to do now is just talk through a couple of the key ideas that we’ve heard emerge and if you can just say, yeah, that sounds right or no, no, no, you totally missed the point of that, I bet what they were trying to say is this. That would be so helpful. So does that sound… are you guys willing to do that? Okay. Great, ‘cause that’s a huge help. So the first big theme that has come out of a lot of these focus groups that we’ve done, and we touched on it a little bit tonight, also… but something that we’ve heard a lot of women say is that, you know, when we ask about, well, why aren’t more trans women on PrEP a lot of women have said, well, trans identity, being trans is not a risk factor for HIV or it’s not a reason to take PrEP. It’s risk behaviors, sexual behaviors that people engage in, so it’s not being trans that puts you at risk; it’s, you know, engaging in unprotected sex or it’s engaging in sex work, or it’s engaging in sex in relationships where there’s not, you know, equal power dynamics. So a lot of what we heard is people saying, well, you’re kind of asking the wrong question. You should be asking about what behaviors is it that makes people at risk for HIV. So I’m curious what, you know… does it sound like we’ve understood that… that concern that people raised about the, you know, the questions that we’re asking in this focus group? Is that something you guys felt when I started asking these questions, like, oh, no, she’s asking the wrong question? I’d love to hear your thoughts on that.

R: Hello?

Mod: I can’t tell who’s talking now. [name redacted]?

R: [name redacted].

Mod: Oops, we keep losing… we keep losing [name redacted]. Um, well, what do you all think?

R: Um, what was the question again? I’m sorry.

Mod: Yeah, so the question is one of the things that we started hearing in these focus groups… you know, I always ask these same sorts of questions, and a lot of women started saying to us, you know, you’re asking about how does me being trans put me at risk for HIV, but it’s not… it’s not being trans that puts a person at risk for HIV, it’s the behaviors that they engage in. I mean we all know there’s certain behaviors that are riskier for HIV, so you should really be asking questions about behaviors that people behave in rather than just asking trans women, you know, oh, tell me about your risk for HIV.

R: Agreed.

Mod: So how does that square with your… with your thoughts on this?

R: I agree, I agree 100%. I agree.

Mod: Okay. Can you say more about that?

R: I feel like… like you said, it doesn’t matter about you being trans, no matter if I’m trans or cis, you know, I can still have… I can still come in contact with HIV, so I feel like it should be more of a topic of, you know, things that you should be doing and how to protect yourself rather than generalizing trans women and HIV.

Mod: Oh, okay, so… and I like how you kind of flipped it there, saying we need to focus not on risk, you know, people are at risk and people shouldn’t do risky things, but flip it and focus on here’s things that everyone can do to protect themselves and that’s what we should really focus on. I like that. I like that. Other thoughts on this?

R: I can agree with that as well. I think it’s important to help give people the tools they need to protect themselves in those kind of situations that they choose to engage with. I do feel it’s also important though to make sure all communities involved have that information. Like how it was discussed before, a lot of trans women aren’t even aware of PrEP so not specifying them as a group at risk isn’t the thing, expressing them as a group that might want to have the tools available for them might be a good way of going about it.

Mod: Okay.

R: But any group that would need it should be looked at.

Mod: Yes, and this is so helpful. This is exactly the kind of sort of, you know, flip because, you know, we’re still talking about the same things. You know, our goal, of course, is to provide information and support people, but it’s important not to frame it as people are at risk and here’s what you need to… but really framing it from everyone deserves to have access to information and our goal is to provide information to people. Alright, we’re done here (laughs). And that’s… that is… that’s exactly the kind of insight that we’re looking for and that really helps… that really helps to think about a way of reframing this and, you know, thinking about a positive impact that this work, that, you know, that Dr. Van Gerwen has worked so hard on and that, you know, that we’re trying to do, that’s a positive way of framing this work. So I really, I appreciate that. Okay. Yeah, okay, you all blew my mind. That’s really… so let’s talk about another one. And this touches on something that [name redacted] kind of talked about when they talked about using the ad or seeing ads on TV and one of the things that we heard a lot in our first couple of focus groups is as soon as you say HIV, as soon as you start talking about HIV prevention or particularly when you start talking about PrEP that immediately goes to men who have sex with men. That’s who we think of, that’s who’s in the ads, that’s who all the billboards are focused at, and so you’re asking us why aren’t trans women more aware of PrEP and of HIV prevention. Well, because it’s all targeted toward men who have sex with men. Does that… does that match with your experiences with the information that you have seen about PrEP or the sort of way that you hear people talk about PrEP? What do you think?

R: For me, again I haven’t been made aware of much PrEP at all, but definitely with HIV in general it is usually targeted to homosexual males who, um, especially when kids are younger and that kind of stuff, it is sort of targeted to them even though almost anyone can get HIV it’s just targeted to those more, groups more due to the history of HIV in general and less on who needs the information now.

Mod: Okay. So, again, it’s that idea that the goal is to provide information for everyone because everyone should have the tools to protect themselves and to have healthy sexual lives, okay?

R: And if there is a group that needs it specifically or is at higher risk they should be looked into and make sure they have the information they need as long as they’re not generalized, and the opposite of romanticized about it.

Mod: Yeah, sort of stigmatized almost?

R: Yeah.

Mod: Yeah, kind of talking about, well, we know that this group is at high risk for HIV… yeah, yeah, that’s useful. Other thoughts on that, about how PrEP is usually kind of talked about in terms of men who have sex with men primarily?

R: Um, yes, and that’s still kind of confusing to me, like [name redacted] was just sayin’, I don’t understand why it has to be targeted to homosexuals when, you know, it can be… it’s for everybody because straight people can contract the same diseases that homosexuals can contract.

Mod: Uh-hum, uh-hum. Yeah, so finding that balance, it sounds like, of making sure that groups who historically have been at risk are getting that information but not like only targeting those groups and certainly not in a way that’s stigmatizing or sort of calling people out as, well, we know you’re likely to be at risk. Okay. That’s… that’s a useful sort of… figuring out how to balance that and provide information in a way that doesn’t really single anyone out. That’s useful to think about. One of the other things that we heard lots of people say in the focus groups is, you know, yeah, we’ve seen ads on TV about PrEP or we’ve seen billboards, but we don’t see transgender women represented in those ads. And that’s something, this idea of representation has been an important theme across all of these focus groups. And so, you know, sort of thinking about the different places that you get information. [name redacted], you talked about using LGBT websites and you found that that was a good source of information. Did you feel like trans women were well-represented on those websites and like when you look at magazines or social media or TV ads do you feel like trans women are a part of the discussion around HIV or are they just missing from that discussion?

R: For me personally when I’m on those websites they’re usually more broad in how they give information. They don’t depict it or have implicit this group or that group; they’re more of just information is straight given. That can be very useful. The main problem with stigmatizing a group to it is then it becomes normalized for us to think that only that group needs it and it becomes abnormal for anyone outside that group to even consider it. So representation matters to normalize people, at least after years of it not being normalized, for people to get in the door and start seeking more information and more help because if it’s normalized no one will feel that it’s wrong or you should hide from it or anything like that.

Mod: Okay, so trying to make it more inclusive so that everyone, yeah, feels like they can access information and stuff. Okay. Other thoughts on that, about just the idea of how we, you know, how we see trans women in the media, how we see representations and how that play into people’s willingness to think about PrEP, for instance?

R: For me, I do not like it. I feel like the representation that they portray trans women to be is a joke. Like it’s always like they’re the laugh, I just don’t like it, I don’t like it at all. I mean not sayin’ that there’s a lot of that, but 75% of what I see it’s always portraying it for me bein’ a trans woman myself, you know, it’s different than how they portray it to be from us seeing it, from us viewing it like on a show or something like that. So I feel like it’s always like we’re supposed to be the laugh and for that that’s why they’ll always assume… no, I just don’t. I could talk about it forever, but no.

Mod: Okay, so that… that’s something we thought about a lot, sort of the complexity of, you know, then how do you… thinking about media messages, how do you target media messages when most of the representations that we have of trans women are not positive, supportive representations, but are sort of laughable, as you’ve said, and so what does that look like? How do you still include trans women in the conversation if the way that we’re representing women’s lives is just not accurate. So, yeah, I don’t… I don’t think… I mean we’re not going to come to clear answers on that, but it is something that we’ve definitely heard across a of these focus groups and so it’s important for us to be thinking about, you know, in terms of next steps or, you know, for the work that we want to do to try to get the word out about PrEP and other HIV prevention opportunities. So I’m glad that we’ve… I’m glad that we’ve heard that and I appreciate you sharing, [name redacted] in particular, that. So the last thing, and I am mindful of time, I know that we’ve only got a few more minutes and I want to make sure that we have time to discuss and debrief when we’re done… the last thing that we’ve heard a lot of trans women say when we talked about PrEP in particular were just sort of concerns about, well, you know, I might be interested in PrEP but I hear that it’s not good for my body or I hear that it might interfere with some of the other medications that I’m taking or it might interfere with my transition for people who are transitioning, and I wonder is that… does that… are we understanding that concern right and are there particular things that we need to be thinking about more, like particular types of information that we could provide to trans women to help them better understand the ways that PrEP might interact and how they can make the best decision for themselves given what their goals are in terms of both, you know, protecting themselves from HIV but also for people who are transitioning to supporting that and not complicating that process? So what do you… what do people think about that? Is that something that’s come up?

R: I haven’t heard much about PrEP in general so I don’t know much of the side effects it may include, but usually from my knowledge, knowing and experience, hormones and medication as a whole can be very… not sensitive, but a very (inaudible) thing for some individuals to talk about and manage, mainly due to how much HRT and transitioning can be stigmatized in the medical area, as it’s gotten better through the years but (inaudible) as a bad thing, as in you’re messing up your body, you’re messing up blah, blah, blah, and once an individual goes through with it they can still have that lingering doubt. And they can feel, okay, I’m stable right now but I don’t want to add anything that could lead to problems or prove those people right or…

Mod: Oh, okay.

R: Or take it one step forward, two steps back, or like they have gone this far and they don’t want it to then in their mind have it taken away from them through another kind of medicine.

Mod: Yeah.

R: That’s kind of two ways I imagine people thinking about.

Mod: Yeah that’s a really a helpful framing. That helps me to understand what people have told us. A lot of people have said my transition is the most important thing right now, you know, living my authentic life, being my true self, that’s what I’m prioritizing right now. And if there are things that might, like you’ve said, one step forward, two steps back, you know… if I’m… if I don’t know exactly how other medications are going to interact with my transition then I’m just not willing to risk that. And you’ve sort of revealed another layer of that, that, you know, the decision to transition, the decision to start HRT is already sort of fraught enough and that there’s just an added layer of (sighs), if this messes things up, then, yeah, okay.

R: And one thing that I’ve seen help individuals as far as to be crystal clear with what it does to the body, the medication itself, because usually when you are talking, when you learn about hormones you usually have to go through every single step and you learn what it does to the body, how it interacts with it and what changes would happen. And usually with that kind of understanding if they’re presented with a new drug as long as it’s defined well enough and clear enough to the individual they’re more willing to accept it or more willing to realize the… how it will actually affect them instead of speculating into the unknown and more about worry about potential harm. Instead they’ll go, okay, so yeah, I already know how HR interacts with my body and I’m told how this medicine will interact, so I can be secure that even if I take it I will be… I’ll be aware of anything that could happen so I could make a more informed decision.

Mod: Yeah, well, and so that points, you know, sort of to the need for… you know, we probably need additional studies of people taking both so that we can speak more to what the common, you know, interactive sort of side effects are, but also that really speaks to the need for providers to be educated and to know how to answer people’s questions because these were questions that we heard come up throughout these focus groups, you know, and people would ask us. And I’m not a medical doctor; Dr. Van Gerwen is, but, you know, and making sure that any providers that are working trans patients really know their stuff and know how to answer these questions rather than just sort of across the board, everyone PrEP, let’s everyone get on PrEP right now, but really being able to talk through people’s concerns about what the interactions are, and talk sensitively. You know, I… I think often times people are like, well, of course you want to protect yourself from HIV, everyone should want to protect themselves from HIV and here I’m offering you something that can protect yourself from HIV, why wouldn’t you take it. But recognizing that there’s, you know, there’s competing… there’s competing needs. There’s more going on that just, you know, preventing potential HIV risk and being sensitive to that and meeting people where they’re at with that.

R: Right. Even presenting them with, um, maybe the provider can’t answer all their questions but having a source to give, like a website that can list multiple things and demonstrate to them what it does is usually pretty helpful because that’s how a lot of transgender women first learn about HTR, is by going on websites and getting the basic information.

Mod: So that’s…

R: And then taking the next step.

Mod: So that’s a resource that trans women are already used to looking to, is websites? Okay, that is really helpful for us thinking about, you know, what sorts of information we can provide and where we can provide that trans women are going to be able to find it and know to… know that it’s factual and evidence-based and supported by, you know, what we know about how HRT and PrEP might interact, and being honest about the things that we don’t know about, too, because that’s important, too. Oh my goodness, you guys, it’s seven o’clock. (laughs) I want to be respectful of time but I cannot thank you enough. This has been exactly what we all needed to hear and such a great way of wrapping up this phase of the work to really hear all of your voices and hear your perspectives. Are there any other things that you need us to know? Need us to hear before we break for the evening?

R: Not for me. Like I said, I liked the statement about doctors being more educated on, you know, just things that trans women will need and different stuff like that.

Mod: Yeah, and that’s why research and focus groups like this are so important because we have to hear from people. We can’t just sit around in our offices and be like I think that people probably think this. No, the only way that we learn is by… well, Dr. Van Gerwen learns because she is a health care provider so she learns when she’s out and about and doing doctor stuff, but for me this is… this is just absolutely invaluable, so I appreciate so much all of you ladies taking the time to talk with us today and we’ll go ahead and break but if there’s anything else that you want to share or say I’ll stay on for a few minutes if you want to say anything else once we close out. But other than that, thanks again for taking the time to talk through some of these topics with me. I can’t tell you how helpful this was. This was a perfect ending to this study, so triumph, YAY!

Mod: Yeah, I want to echo that, I thank you all so much. I’ve been here the whole time; I’ve just been on mute. So, yeah, I echo everything Ela said, you know, the reason we do this is so that we can hear from the people we’re taking care of so we can take care of y’all better. And so the perspectives and the insights that we’ve gotten from all of these groups is going to go into future work to do better things in the sexual health space to help the trans women in our area live, you know, sex-positive safe, healthy lives. So that is our goal and this is one of the starting points, so, again, thanks, everybody. A little business, we need to reimburse everyone for your time as that’s very important. So I have Visa gift cards for y’all but I will need to get with each of you individually about how to exchange those, so you’ll be hearing from me in the next few days about making plans for that. As Ela mentioned, this is the last part of this study, but we are hoping to do a few more focus groups in this general space, so if you have people who you know who might be interested in participating in something like this, maybe about something else in the sexual health realm, you can receive additional reimbursement for referring them, so just keep that in mind. I think I talked to all of you about that when I had the initial call with each of you. So, again, I’ll be in touch with everybody; I have all of your contact information, and again, thanks so much. I’ll put my email in the chat. I think you all have it but in case anybody needs to get in touch with me I’ll put my email in the chat.

Mod: Alright, well, let’s go ahead and break for the night, but thank you, ladies, again. This was just a great way-…

END OF RECORDING
